# RUNX1‐Mediated Hypoxic Signaling Impairs Myogenesis and Promotes Fibrosis in Myopathies

**DOI:** 10.1002/advs.78533

**Published:** 2026-10-31

**Authors:** Liang Zhao, Taijiang Hou, Lin Zhang, Feng Gao, Tong Xing

**Affiliations:** ^1^ College of Animal Science and Technology Nanjing Agricultural University Nanjing Jiangsu China; ^2^ College of Animal Science and Technology State Key Laboratory of Meat Quality Control and Cultured Meat Development Nanjing Agricultural University Nanjing Jiangsu China

**Keywords:** fibrosis, hypoxia, myogenic repair, myopathy, RUNX1

## Abstract

Compromised myogenic repair with enhanced fibrosis is common in myopathies, including Wooden Breast (WB) myopathy in broilers, but the underlying mechanism of their co‐occurrence remains elusive. Using WB myopathy as a model, we investigated the common pathological origins of these co‐occurring features. Our single‐nucleus RNA sequencing results showed enhanced oxidative stress and profibrotic remodeling of myofibers, increased proliferation but decreased differentiation of satellite cells (SCs), and aggravated differentiation of fibroblasts (FBs) in WB samples. Differential gene expression and pseudotime trajectory analysis further identified RUNX1 as a key regulator for both SCs and FBs, which was further supported by gene functional studies in respective cell culture models. Moreover, reduced blood flow and oxygen supply may indicate a hypoxic state in WB muscle, which was associated with enhanced RUNX1 expression. Consistently, coordinately enhanced *HIF1A* and *RUNX1* expressions were found in differentiated FBs of human muscles and in muscles with diseases. Collectively, our study found that hypoxia‐associated upregulation of *RUNX1* disrupted the myogenic progression of SCs and exacerbated fibrotic differentiation of FBs, contributing to pathological degeneration in myopathies. Targeting RUNX1 may therefore represent a potential therapeutic strategy for prevention and treatment, which warrants future in vivo investigations.

## Graphical abstract

Myofiber overgrowth was associated with reduced blood flow and a potential hypoxic state in broiler breast muscle, accompanied by increased RUNX1 expression. Elevated RUNX1 impaired myogenic regeneration, promoted fibrosis, and disrupted the signaling niche required for efficient muscle repair, contributing to pathological degeneration in Wooden Breast (WB) myopathy. Comparative analyses further suggest that the HIF1A/RUNX1 regulatory axis may be relevant to muscle diseases in humans and mice, highlighting RUNX1 as a potential therapeutic target. The TOC figure was created with BioGDP.com and Adobe Illustrator.

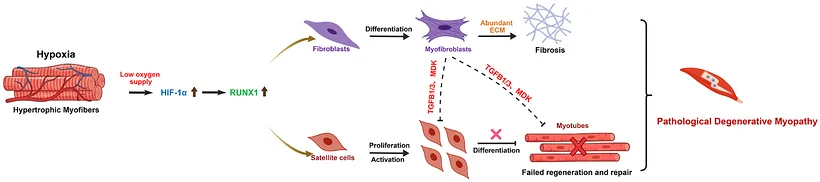

## 1 Introduction

As the largest organ in humans and animals, skeletal muscle not only determines physical mobility and mechanical strength but also plays essential roles in systemic metabolism, including glucose homeostasis and lipid oxidation. Consequently, skeletal muscle dysfunction caused by injury, disease, or aging can lead not only to impaired movement but also to metabolic disorders such as insulin resistance and obesity [1]. In myopathies of various etiologies, compromised myogenic regeneration is typically accompanied by excessive collagen accumulation. Studies have shown that elevated fibrotic activity further exacerbates the myogenic repair process. Therefore, enhancing myogenic repair while preventing fibrosis is key to the prevention and treatment of myopathies. Wooden Breast (WB) is a globally prevalent myopathy affecting the pectoralis major (PM) muscles of fast‐growing, modern broiler chicken breeds. Affected muscles typically exhibit a turbid, viscous coating on the outer surface, small intramuscular hemorrhages, and marked rigidity and hardness. Histologically, WB‐affected muscle tissue is characterized by degenerated myofibers accompanied by increased deposition of fibrotic tissue and infiltration of inflammatory cells [2, 3]. These abnormalities compromise meat quality and cause substantial economic losses to the broiler industry. Nevertheless, the etiological factors underlying WB development remain poorly understood.

Muscle regeneration after injury relies on the myogenic capacity of satellite cells (SCs), which are the adult tissue stem cells residing between the basal lamina and sarcolemma of muscle fibers [4, 5]. Under resting conditions, SCs remain quiescent, but they can quickly re‐enter the cell cycle following injuries. Through a well‐orchestrated process of activation, proliferation, and differentiation, SCs ultimately transform into new myofibers or fuse with existing ones to facilitate repair. Only a subset of self‐renewing cells can resist differentiation during muscle injury and will return to quiescence upon completion of muscle regeneration, thereby replenishing the SC pool and priming the tissue for future injuries [6]. Disruption of this tightly regulated myogenic process in myopathies, neuromuscular disorders, and muscles with metabolic disease has been reported to lead to compromised muscle regeneration. For example, muscles affected by Duchenne muscular dystrophy exhibit defective cell renewal alongside increased activation and differentiation, a combination that contributes to exhaustion of the SC pool and ultimately leads to severe muscle deterioration [7]. Similarly, in broiler breast muscles, SC numbers decline, and their inherent proliferative and differentiation capacities diminish with increasing age and size [8]. Therefore, elucidating the mechanisms underlying disrupted myogenic progression of SCs in broilers is critical for understanding muscle degeneration in WB.

In addition to the disrupted function of SCs and compromised repair of myofibers, connective tissue fibrosis is another key pathological feature in myopathies [9, 10]. Fibrosis arises from the injury‐induced activation of fibroblasts (FBs) and their subsequent differentiation toward a fibrotic phenotype, known as the myofibroblast [11]. Compared to quiescent fibroblasts, myofibroblasts exhibit larger cell volume and distinct structural features, notably α‐smooth muscle actin (α‐SMA)‐rich actin bundles, which enhance their migratory capacity and contractile force generation at injury sites [12]. The dynamic transition of fibroblasts to myofibroblasts leads to overproduction of extracellular matrix (ECM) components, particularly type I collagen and fibronectin, as well as contraction of actomyosin‐based stress fibers. In a healthy regenerating microenvironment, myofibroblast activation is transient, with cells undergoing apoptosis or returning to quiescence after efficient repair. Conversely, their persistent activation under pathological conditions leads to excessive fibrosis [13]. For example, WISP1 secretion from FBs under healthy conditions promoted the amplification and commitment of SCs, while loss of WISP1 due to fibrotic differentiation of FBs delayed muscle repair [14]. Previous studies in our laboratory also found enhanced deposition of ECM proteins, including collagen I and fibronectin, in the PM muscle of WB‐affected broilers, which was associated with activation of the TGF β1/Smad signaling pathway [15]. Thus, preventing the transition of FBs to myofibroblasts would not only decrease fibrotic connective tissue accumulation but also promote muscle repair efficiency.

Both the myogenic capacity of SCs and the fibrotic differentiation potential of FBs are heavily affected by signals from the skeletal muscle niche [16, 17, 18]. In fast‐growing broilers with high breast muscle yield, myofiber enlargement outpaces capillary development, leading to insufficient vascularization [19]. This restricted blood flow impairs nutrient and oxygen delivery to myofibers, resulting in stress and local hypoxia [20]. The transcriptional regulators, hypoxia‐inducible factors (HIF‐1α/HIF‐2α), become activated in response to hypoxia [21]. Double knockout of HIF1α/2α in myoblasts reduced self‐renewal but enhanced differentiation under hypoxic conditions in an injured mouse model [22]. Moreover, hypoxic signaling and enhanced expression of HIF‐1α have also been observed in different models of skeletal muscle fibrosis [20, 23, 24]. Crucially, the impact of hypoxia on tissue repair is not uniformly deleterious; rather, it elicits divergent adaptive or pathological responses dictated by its temporal and qualitative severity [25]. Recent literature highlights hypoxia‐inducible factor‐1α (HIF‐1α) as a master regulator that orchestrates cellular adaptation via the modulation of glycolysis, angiogenesis, and stress‐response pathways. Under progressive hypoxia, differential regulation of HIF‐1α stability by oxygen‐dependent hydroxylases enables cells to selectively activate downstream programs required for survival and metabolic adaptation. Therefore, dysregulated hypoxic signaling may disrupt the balance between regeneration and fibrosis by altering cellular metabolism and cell fate decisions [26]. However, the functional mechanism by which hypoxia‐induced disturbances in myogenesis and fibrosis contribute to WB myopathy has not yet been systematically studied.

In this study, we investigated the mechanisms underlying the co‐occurrence of impaired myogenesis and exacerbated fibrosis in skeletal muscle, using broilers with WB myopathy as a model. Utilizing high‐resolution single‐nucleus RNA‐seq (snRNA‐seq) combined with in vivo and in vitro studies, we found that reduced perfusion and the accompanying potential hypoxic state in WB muscle were associated with increased RUNX1 expression in both SCs and FBs. Gain‐ and loss‐of‐function experiments further showed that RUNX1 promotes proliferation while suppressing the differentiation of SCs, and aggravates the fibrotic differentiation of FBs. In re‐analysis of human and mouse samples from publicly accessible datasets, enhanced RUNX1 expression was also consistently observed in differentiated FBs and muscles with various myopathies. Therefore, we propose that RUNX1 is a critical regulator of the pathological development of myopathies, and that targeting RUNX1 may serve as a potential therapeutic strategy.

## 2 Materials and Methods

### 2.1 Ethics Statement

All animal management and experimental procedures were approved by the Institutional Animal Care and Use Committee of Nanjing Agricultural University under protocol number SYXK 2021–0014. All procedures involving animals were conducted in accordance with the institutional guidelines for the care and use of experimental animals.

### 2.2 Experimental Broiler Chickens Selection and Sample Collection

All birds were 42‐day‐old male Arbor Acres broilers from the same commercial batch, raised in identical layered‐cage conditions and fed the same commercial feed with free access to food and water. To ensure randomization during animal selection, an initial pool of 300 broilers from the same batch was randomly selected from the farm for in vivo evaluation. Based on Papah et al.’s [27] preliminary WB myopathy assessment criteria, a total of 120 broilers were selected and marked, comprising 60 broilers preliminarily identified as normal and 60 affected by WB. The selected broilers were slaughtered via electrical stunning followed by exsanguination via the carotid arteries. Subsequently, based on the histological classification criteria for pectoral muscle (normal muscles showing tightly packed polygonal fibers without degenerative changes and severe WB muscles showing polyphasic myodegeneration, necrosis, inflammatory‐cell infiltration, and fat/connective‐tissue accumulation), 8 confirmed normal muscles and 8 confirmed severe WB muscles were selected as the final experimental groups (*n* = 8 birds per group) to ensure the accuracy of the study [28]. For snRNA‐seq analysis, equivalent amounts of PM muscle tissues from the 8 independent animals within each group were physically pooled before nuclei isolation and library construction, generating one pooled sequencing library for the CON group and one pooled sequencing library for the WB group. The pooled library therefore served as the experimental unit for the snRNA‐seq dataset; accordingly, sequencing‐derived comparisons were not interpreted as condition‐level inference based on independent biological replicates. The remaining tissues from individual animals were collected separately, flash‐frozen in liquid nitrogen, and stored at −80°C for biochemical analysis. Concurrently, samples from the same region were cut to specific dimensions and fixed in either 4% paraformaldehyde or 2.5% glutaraldehyde solution for morphological observation.

### 2.3 Single‐Nucleus RNA Sequencing (snRNA‐seq) Analysis

Normal (CON) and wooden breast (WB)‐affected PM muscle tissues were dissociated into single‐nucleus suspensions (cell viability > 80%) from pooled samples for library construction using the 10X Genomics Chromium system, followed by sequencing on an Illumina NovaSeq 6000 platform. One sequencing library was generated for each condition (CON and WB). Raw sequencing data were processed using Cell Ranger (version 4.0.1) and aligned to the chicken reference genome (GRCg7b) to generate feature‐barcode matrices. Subsequent quality control was performed using the Seurat R package (version 3.2.0), retaining cells with > 400 unique molecular identifiers (UMIs), < 50% mitochondrial gene transcripts, and appropriate gene detection thresholds, followed by LogNormalize data normalization. Based on highly variable genes, principal component analysis (PCA, utilizing the top 10 PCs) and Uniform Manifold Approximation and Projection (UMAP) were sequentially applied for dimensionality reduction, cell clustering, and visualization, with distinct cell populations annotated utilizing canonical marker genes. Differentially expressed genes (DEGs) were identified via the Wilcoxon rank‐sum test (thresholds: |log2FC| ≥ 1 and adjusted *P* < 0.05) and subsequently subjected to GO/KEGG pathway enrichment and Gene Set Enrichment Analysis (GSEA) using the clusterProfiler package. Finally, pseudotime trajectory analysis was conducted using Monocle3, which performed dimensionality reduction and cell ordering based on highly variable genes to construct and visualize inferred developmental lineages from cross‐sectional data.

### 2.4 Microscopic Analysis

Ultrastructural observations were performed using the methods of Velleman et al. [29] for fixation, embedding, and sectioning. Finally, transmission electron microscopy (Hitachi H‐7650, Japan) was used to observe the ultrastructural changes of muscle samples. Masson staining and Sirius Red staining were performed according to the procedures described by Xing et al. [15] and Li et al. [30], respectively, for paraffin embedding, sectioning, and staining, and images were obtained using an optical microscope (BX50, Japan). Histological classification and all quantitative image analyses were performed by an investigator blinded to group assignment.

### 2.5 Cell Culture

Primary satellite cells were isolated from the PM muscles of 12‐day‐old Arbor Acres broiler embryos, expanded, and subsequently induced to differentiate. Each independent biological replicate for satellite‐cell experiments was generated by pooling cells isolated from 2 to 3 embryos. Additionally, the chicken embryo fibroblast cell line DF‐1 (SNL‐006, Wuhan Sunncell Bio‐Technology Co., Ltd, Wuhan, China, RRID: CVCL_0570) was cultured and treated with 10 ng/mL TGF‐β1 (HY‐P7118, MedChemExpress, USA) for 24 h to induce differentiation. Comprehensive protocols regarding cell isolation, culture conditions, differentiation, and induction are detailed in the Section S1.

### 2.6 siRNA, Plasmids, and Transfection

Both the pcDNA3.1‐RUNX1 overexpression plasmid and the empty pcDNA3.1(+) control vector were designed and synthesized by Shanghai GenePharma Co., Ltd. Three specific siRNAs targeting RUNX1 and a non‐targeting scrambled control siRNA were designed using the Invitrogen Block‐iT RNAi Designer (Thermo Fisher Scientific) and synthesized by Shanghai Sangon Biotech Co., Ltd. The detailed sequence information is listed in Tables S1 and S2, respectively. According to the manufacturer's instructions, Lipofectamine 3000 (L3000008, ThermoFisher) was used to transiently transfect the plasmids and siRNAs into primary satellite cells and fibroblasts, respectively.

### 2.7 Protein Extraction and Western Blot

Western blot analysis was performed as described previously [31, 32]. The primary antibodies used included RUNX1 (25315‐1‐AP, 1:5000, RRID: AB_2880026), α‐SMA (14395‐1‐AP, 1:2000, RRID: AB_2223009), collagen I (14695‐1‐AP, 1:2500, RRID: AB_2082037), MyOG (26762‐1‐AP, 1:3000, RRID: AB_3669566), and MyOD (18943‐1‐AP, 1:2000, RRID: AB_10603467), all purchased from Proteintech (Wuhan, China). The primary antibody against HIF‐1α (3716, 1:1000, RRID: AB_2116962) and the secondary antibody (7074, 1:3000, RRID: AB_2099233) were obtained from Cell Signaling Technology. Protein bands were visualized using a SuperFemto ECL Chemiluminescence Kit (E423‐02, Vazyme, Nanjing, China) and imaged with a ChemiDoc Imaging System (Tanon Science & Technology, Shanghai, China).

### 2.8 Quantitative Real‐Time PCR

Total RNA was extracted from cells using RNAiso Plus reagent (9108, Takara, Dalian, China). The obtained RNA was reverse‐transcribed into cDNA using the ABScript Neo RT Master Mix for qPCR with gDNA Remover (RK20433, ABclonal, Wuhan, China). Real‐time quantitative PCR was performed using the 2X Universal SYBR Green Fast qPCR Mix (RK21203, ABclonal, Wuhan, China). The PCR cycling conditions were as follows: 95°C for 30 s; 40 cycles of 95°C for 10 s, 60°C for 30 s, 95°C for 15 s, and 60°C for 1 min; followed by 95°C for 15 s. The RT‐qPCR results were analyzed using the 2^(−ΔΔCt) method. Each biological replicate was assayed in 3 technical replicates, and the mean of the technical replicates was used as the value for that biological replicate. All the primers used are listed in Table S3.

### 2.9 Immunofluorescence Staining

The procedure was performed according to a previously described method [33]. Briefly, cells were probed with specific primary antibodies, followed by incubation with corresponding secondary antibodies (A1104, Beyotime, China). Cell nuclei were counterstained with DAPI (C1002, Beyotime, China), and fluorescence images were acquired using a laser scanning confocal microscope (Nikon T300, Japan).

### 2.10 Cell Proliferation Assays

Cell viability was assessed using a Cell Counting Kit‐8 (CCK‐8, C0038, Beyotime, China) assay [34]. Briefly, cells were seeded in 96‐well plates and, at 24 h after transfection, 10 µL of CCK‐8 solution was added to each well and incubated at 37°C for 1 h. The absorbance was then measured at 450 nm using a microplate reader.

Cell proliferation was assessed by a 5‐ethynyl‐2’‐deoxyuridine (EdU) incorporation assay [35]. Cells were seeded in 12‐well plates and, at 24 h after transfection, were incubated with growth medium containing 10 µM EdU (C0071S, Beyotime, China) for 3 h. Cells were then fixed and stained according to the manufacturer's instructions, and cell nuclei were counterstained with DAPI (C1002, Beyotime, China). EdU‐positive (DNA‐synthesizing) cells were visualized under a laser scanning confocal microscope (Nikon T300, Japan) and quantified using ImageJ to determine the proportion of proliferating cells. All quantitative image analyses were performed by an investigator blinded to the treatment group.

### 2.11 Blood Analysis and Blood Flow Measurement

Following established protocols [36], wing vein blood samples (1 mL) were collected and immediately analyzed for blood gas and electrolyte parameters including oxygen partial pressure (pO_2_), oxygen saturation (sO_2_), partial pressure of carbon dioxide (pCO_2_), total carbon dioxide (TCO_2_), hematocrit (HCT), hemoglobin (Hb), sodium (Na^+^), potassium (K^+^), and ionized calcium (Ca^2^^+^) using the epoc Blood Analysis System equipped with a BGEM test card. After ether anesthesia, the breast skin of the broilers was incised to completely expose the PM muscle. Real‐time blood flow in the cranial region of the PM muscle was then monitored and recorded using a laser speckle contrast imaging system (Moor Instruments, UK). The detailed results of these measurements are presented in Figure S3.

### 2.12 Hypoxia Treatment

Cells were seeded into 6‐well plates and cultured under normoxic conditions for 24 h. Upon reaching 80% confluence, cells were designated as either the control group (CON) or the hypoxia group (HYP). After replacing the medium with fresh culture medium, the CON group was maintained in a normoxic incubator, while the HYP group was transferred to a tri‐gas hypoxia incubator set to 37°C, 1% O_2_, 5% CO_2_, and 94% N_2_.

### 2.13 Cross‐Species Analysis of Publicly Available Human and Mouse Skeletal Muscle Cell RNA‐seq Datasets

The snRNA‐seq datasets for human skeletal muscle cells were obtained from the UCSC Cell Browser (https://cells.ucsc.edu/?ds=muscle‐cell‐atlas and https://cells.ucsc.edu/?ds=muscle‐ibm+myonuclei). The transcriptome sequencing or microarray datasets of skeletal muscle tissues from 9 patients with myotonic dystrophy and 4 diseased mice were obtained from the DiSignAtlas (http://www.inbirg.com/disignatlas). The UMAP plots and feature plots generated in this study were plotted and modified using the above websites and the CNSknowall website (https://www.cnsknowall.com/). The original sequencing data were also published and made accessible to the public by the authors [37, 38].

### 2.14 Cell–Cell Communication Analysis

Intercellular communication was inferred with the CellChat R package (version 2.0.2) using the normalized expression matrix and the cell‐type annotations of the 10 annotated clusters of the CON and WB libraries, with CellChatDB as the reference database [39]. Since the ligand‐receptor annotations in CellChatDB are mammalian‐derived, the human gene symbols were converted to their chicken homologous symbols using the babelgene package (version 22.9), which maps orthologs based on the OrthologsMatrix of the NCBI HomoloGene database. The standard CellChat pipeline was applied, comprising identifyOverExpressedGenes, computeCommunProb (law of mass action), computeCommunProbPathway, and aggregateNet, followed by outgoing and incoming signaling pattern analysis. Communication probabilities were estimated by the trimean method, which normalizes for differences in cell group size, and significance was assessed by the default permutation test with a threshold of *p* < 0.05. Differential signaling between CON and WB was examined with netVisual_diffInteraction and rankNet, with a focus on pathway‐level information flow rather than raw interaction counts because of the different cellular composition of the two groups. Because ortholog mapping only allows conserved ligand‐receptor pairs to be evaluated, the results are presented as predictions of putative interactions rather than as evidence of signaling in vivo.

### 2.15 Statistical Analysis

All data were analyzed using SPSS software (version 27.0, IL, USA) and visualized using GraphPad Prism (version 9.5.1, CA, USA). Throughout the study, n denotes independent biological replicates (independent birds for in vivo analyses; independent cell‐culture experiments for in vitro analyses). Data are presented as mean ± SEM, and the statistical details for each experiment are provided in the corresponding figure legends. The normality of data distribution was assessed using the Shapiro‐Wilk test. For comparisons between two groups, a two‐tailed unpaired Student's t‐test was performed. One‐way ANOVA with Tukey's multiple comparisons was used for comparisons between multiple groups. Significance was considered at *p* < 0.05.

## 3 Results

### 3.1 snRNA‐seq Reveals Cellular Composition Differences Between Samples With or Without Wooden Breast Myopathy

To gain a comprehensive single‐nucleus resolution of the cellular composition differences, snRNA‐seq was performed on samples from control (CON) and WB broilers. A total of 11 791 cells passed quality control with a median gene number of 786. Using UMAP analysis (Figure 1A), we annotated 10 cell types, including macrophages (Mac), two types of myofiber nuclei (Myo1 and Myo2), fibroblasts (Fibro), satellite cells (SCs), T cells, endothelial cells (Endo), B cells, neurons (Neu), and adipocytes (Adi). Among these cell types, noticeably increased proportions were observed for Myo2 and T cells, while the proportions of Myo1, Fibro, SCs, and Endo decreased (Figure 1B), suggesting cell type‐specific differences caused by WB myopathy. Characteristic marker gene expressions for each cell type were listed in Figure 1C. The identity of each cell type was further verified by enrichment analysis of the top 100 marker genes (Figure 1D). The results showed that Myo1 was characterized by muscle cell development and cytoskeleton organization; Myo2 was characterized by muscle system process and respiratory electron transport chain; SCs were characterized by regulation of skeletal muscle tissue development; and Fibro was characterized by extracellular matrix organization. Therefore, our snRNA‐seq analysis successfully identified the main cell populations in the breast muscle, which provided valuable insights for further analysis of their pathological changes caused by WB myopathy.

**FIGURE 1:**
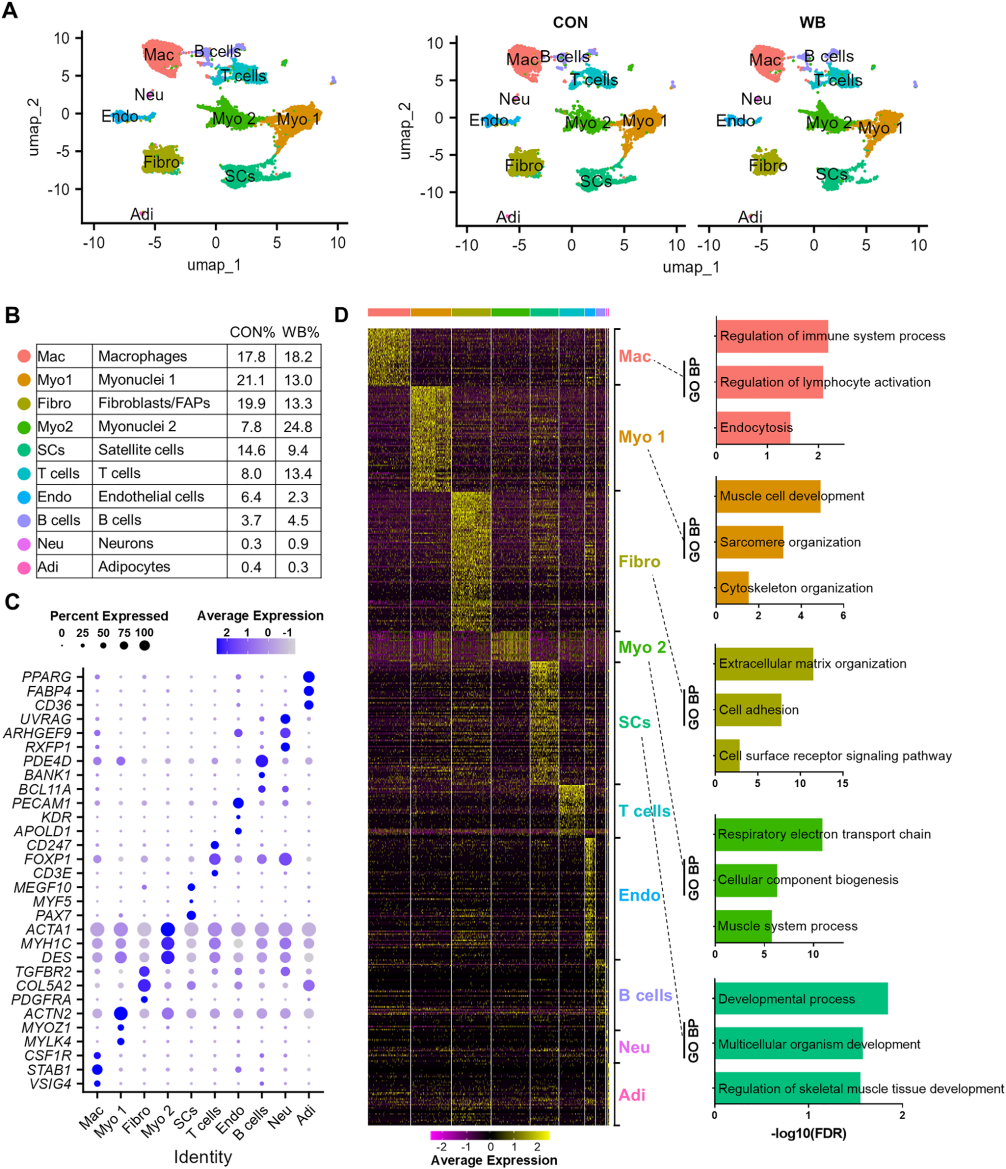
Single‐nucleus atlas of CON and WB muscle. (A) UMAP visualization showing cell clusters identified by unsupervised clustering. (B) The proportional abundance of each cell population. (C) Expression patterns of marker genes used for cell type annotation. (D) Heatmap of DEGs in each cell cluster and the top 3 significantly enriched GO terms for key cell clusters.

### 3.2 Wooden Breast Myopathy Induces Inflammatory, Oxidatively Stressed, and Profibrotic Remodeling of Myofibers

The cell types of myofibers (Myo1 and Myo2) were extracted in silico for further analysis to understand WB‐induced changes in myofiber degeneration. The proportion of Myo2 was markedly higher in the WB group, whereas the proportion of Myo1 was higher in the CON group (Figure 2A). GSEA was conducted to reveal transcriptomic differences between these two myofiber types. The up‐regulated terms in Myo2 include aerobic electron transport chain, cellular response to superoxide, collagen fibril organization, and inflammatory response, indicating enhanced oxidative stress, profibrotic secretion, and inflammation in Myo2 (Figure 2B). In contrast, the down‐regulated terms in Myo2 were more related to normal physiological function of myofibers, such as amino acid and ion transport, neuromuscular synaptic transmission, and sarcomere organization, suggesting a disruption of homeostasis after myofiber remodeling (Figure 2C). Consistently, histological analysis of muscle sections showed significant necrosis and dissolution of myofibers, with vacuolation and swollen mitochondria, and necrotic areas replaced by connective tissue (Figure 2D).

**FIGURE 2:**
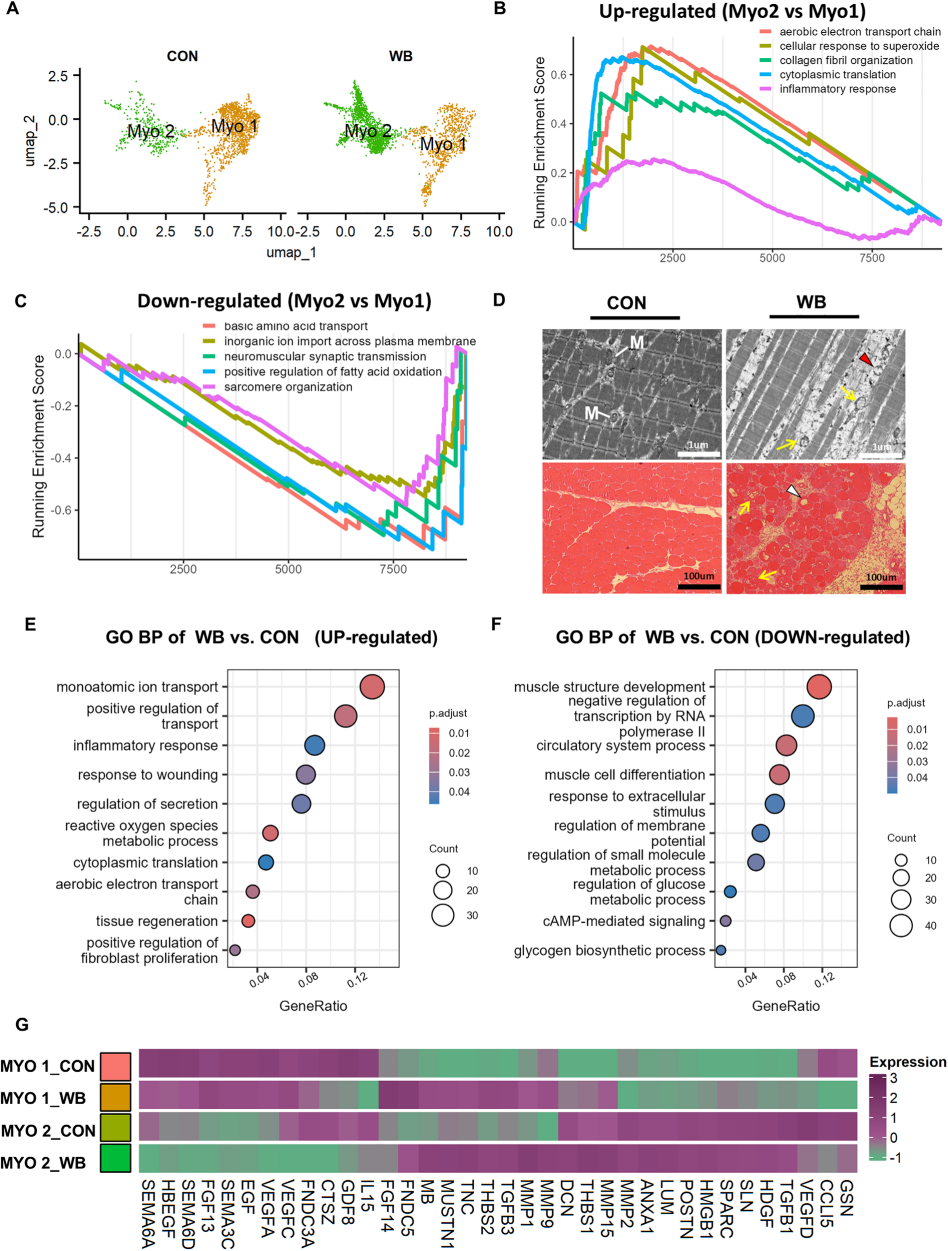
Transcriptional profiles of myonuclei and structural alterations of myofibers in CON and WB muscle. (A) UMAP visualization showing the Myonuclei clusters from CON and WB muscle. (B‐C) GSEA plot for upregulated and downregulated genes in the Myonuclei 2 cluster. (D) Representative images of ultrastructural and Masson staining of CON and WB muscle. Scale bars: 1 µm and 100 µm. (E, F) GO analysis of upregulated and downregulated genes between CON and WB muscle. (G) Heatmap showing the expression levels of representative factors synthesized and secreted by the Myonuclei in CON and WB muscle.

We further compared differentially expressed genes (DEGs) between WB and CON samples (combining Myo1 and Myo2) and conducted GO analysis (Figure 2E,F). Consistent with the above findings, GO terms related to oxidative stress, inflammation, and profibrotic features were also enriched in WB myofibers (Figure 2E), whereas down‐regulated terms were more associated with metabolism and structural maintenance (Figure 2F). DEGs related to myofiber secretion were shown in Figure 2G. The expression of *FNDC5*, *MB*, *MUSTN1*, *TNC*, *THBS2*, *TGFB3*, *MMP1*, and *MMP9* was enhanced in the WB group for both Myo1 and Myo2, whereas the expression of *MMP2*, *ANXA1*, *LUM*, *POSTN*, *HMGB1*, *SPARC*, *SLN*, *HDGF*, and *TGFB2* was enhanced in Myo2 for both CON and WB groups (Figure 2G). In summary, WB induced remodeling of myofibers toward a more inflammatory, oxidatively stressed, and profibrotic state, which contributes to a profibrotic microenvironment in muscle.

### 3.3 Wooden Breast Myopathy Disrupts the Myogenic Progression of Satellite Cells and Increases the Expression of RUNX1

The cluster of SCs from both CON and WB groups was isolated in silico and re‐grouped into four subtypes according to classical markers related to sequential myogenic progression: quiescent (Qui), activated (Act), proliferative (Pro), and differentiating (Diff) SCs (Figure 3A,B). The proportions of Qui, Pro, and Diff decreased in the WB group, while that of Act was markedly higher, suggesting increased activation but decreased proliferation and differentiation of SCs in the WB group (Figure 3C). Comparison of the transcriptomes of Act versus Qui also enriched GSEA terms related to mitotic division of cells in the Act cluster (Figure 3D). Furthermore, cell cycle analysis showed that SCs from the WB group exhibited a higher proportion of cells in the S phase and a corresponding decrease in the G1 phase compared to the CON group, suggesting enhanced G1/S transition and cell‐cycle progression (Figure 3E,F). Taken together, these findings suggest that WB promoted the early activation and cell‐cycle entry of SCs, accompanied by enhanced early proliferative activity, though the relative ratios of SCs in the Pro and Diff states were reduced.

**FIGURE 3:**
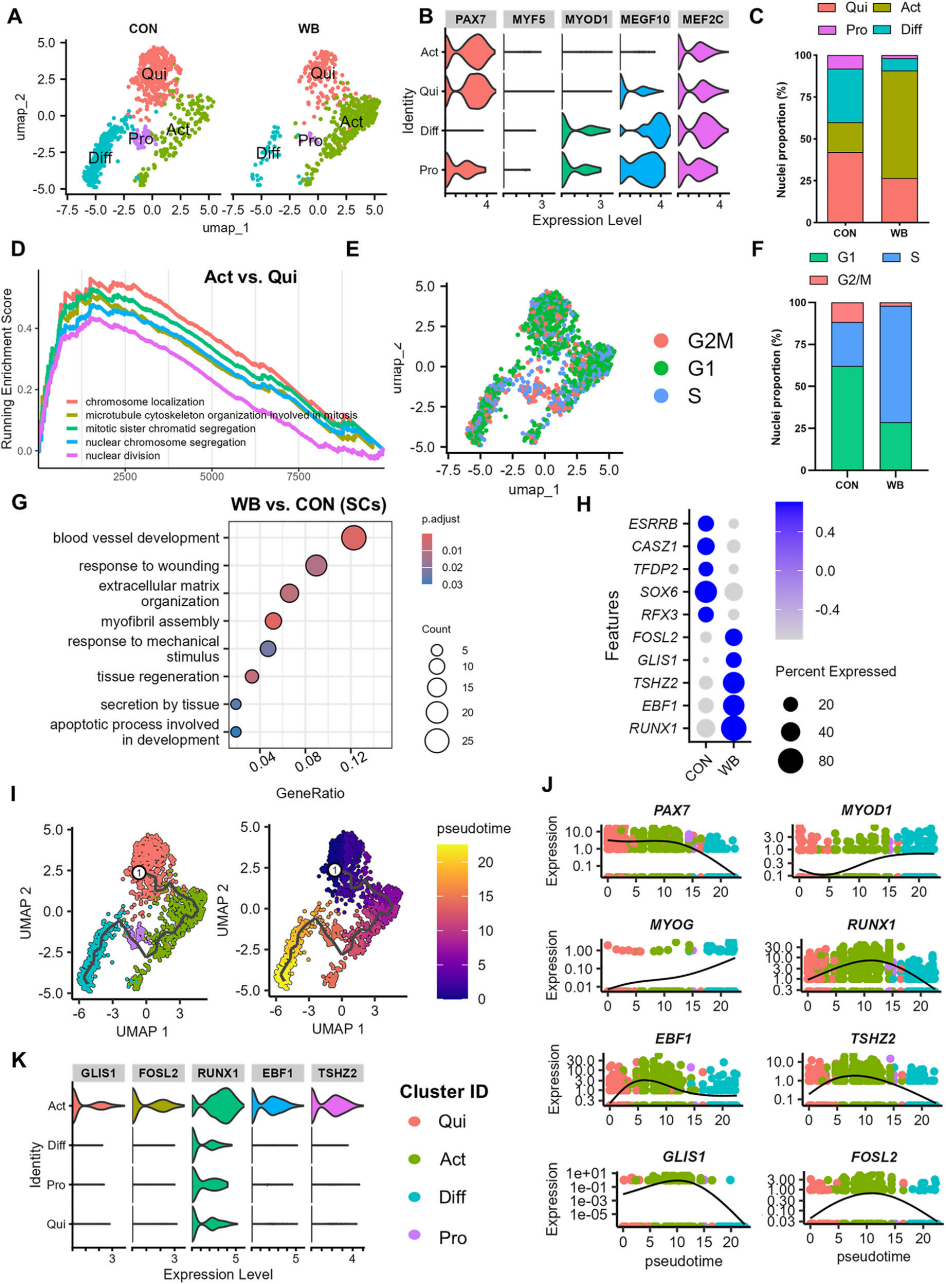
Transcriptional profiling of satellite cells (SCs) in CON and WB muscle. (A) UMAP visualization of SCs clusters in CON and WB muscle. (B) Violin plots showing the expression of marker genes in each SCs cluster. (C) The relative proportions of cell nuclei in each SCs cluster. (D) GSEA analysis of DEGs between the Act and Qui clusters. (E) UMAP visualization of SCs grouped by cell cycle marker genes. (F) The relative proportions of different cell cycle states between the CON group and WB group. (G) Bubble plot of GO enrichment analysis for DEGs between SCs from CON and WB muscle. (H) Bubble plot of the top five highly expressed genes in SCs from CON and WB muscle. (I) Pseudotime trajectory analysis of SCs. (J) Dynamic expression changes of highly expressed transcription factors and SCs marker genes. (K) Violin plot shows the expression levels of highly expressed transcription factors in each SCs cluster.

Further GO analysis of DEGs in SCs showed that biological processes related to extracellular matrix organization, response to mechanical stimulus, myofibril assembly, tissue regeneration, pro‐fibrotic secretion, and apoptosis were enhanced in the WB group (Figure 3G). Among the DEGs, the expression of the top 5 upregulated (*RUNX1*, *EBF1*, *TSHZ2*, *GLIS1*, *FOSL2*) and downregulated (*RFX3*, *SOX6*, *TFDP2*, *CASZ1*, *ESRRB*) transcription factors (TFs) was shown in Figure 3H. Then, a pseudotime trajectory path of myogenesis was constructed using the R package Monocle 3 (Figure 3I). The gradient decrease in *PAX7* expression and the increased expression of *MYOD1* and *MYOG* supported the inferred ordering of this myogenic trajectory. Moreover, the expression of *RUNX1*, *EBF1*, *TSHZ2*, *GLIS1*, and *FOSL2* showed an early increase followed by a decrease, suggesting important functions of these TFs in early myogenic activation (Figure 3J). Further analysis of gene expression patterns in SC subclusters also revealed the highest expression of these genes in the Act subtype (Figure 3K). Together, these data suggest an important function of *RUNX1* in balancing the early activation versus later differentiation of SCs, which awaits further verification.

### 3.4 Wooden Breast Myopathy Induces Enhanced Fibrotic Differentiation of Fibroblasts Accompanied by Increased RUNX1 Expression

The excessive transition of fibroblasts (FBs) to myofibroblasts leads to the accumulation of connective tissue fibrosis in diseased or disordered muscles. Compared to the CON group, up‐regulated DEGs in the WB group enriched multiple KEGG terms related to the extracellular matrix organization, collagen metabolic process, and fibrotic differentiation of FBs (Figure 4A). In contrast, the down‐regulated DEGs enriched terms mostly related to synaptic exocytosis and the tyrosine kinase signaling pathway (Figure 4B).

**FIGURE 4:**
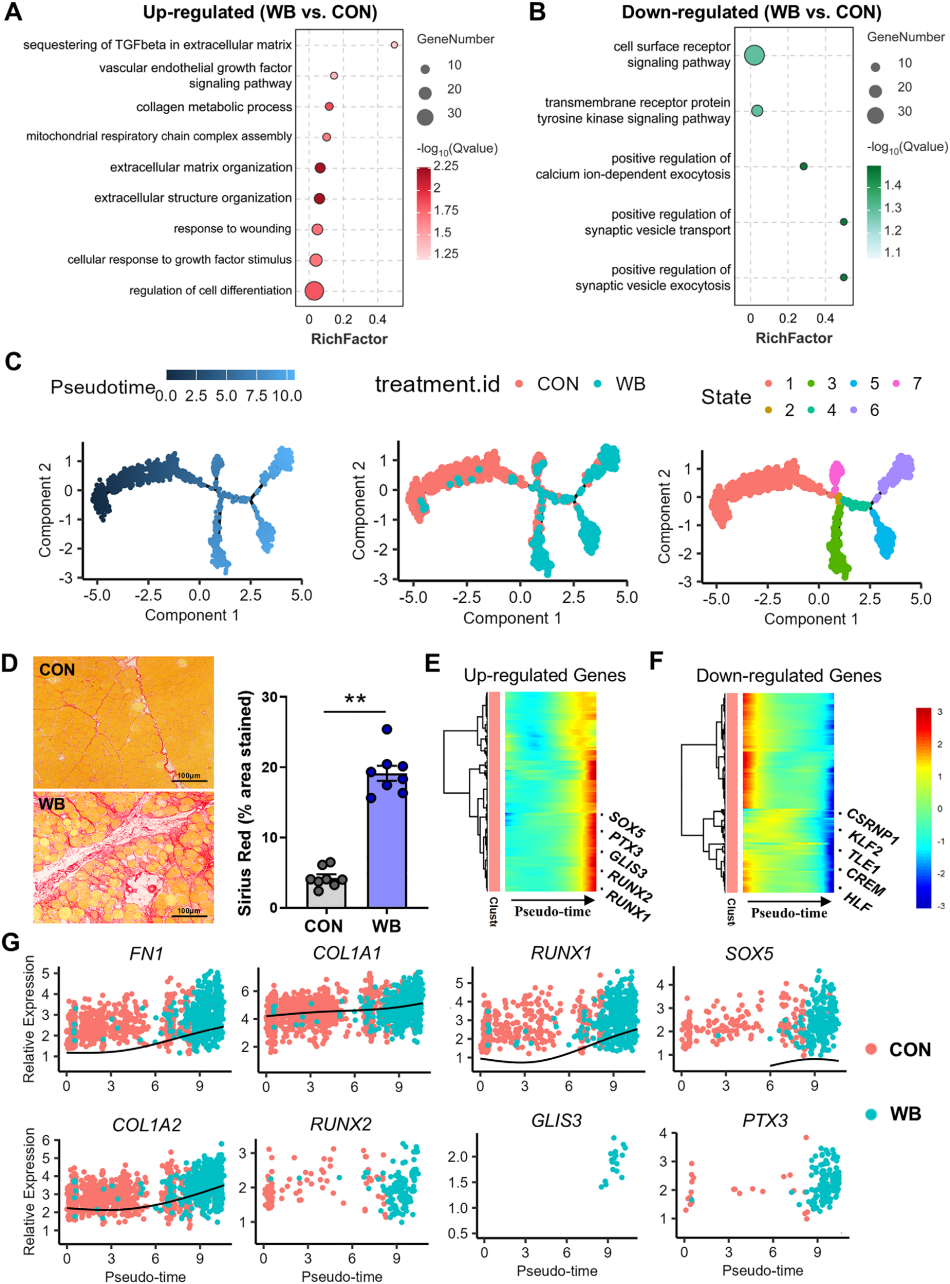
Alterations in the transcriptional profile of fibroblasts between CON and WB muscle. (A, B) Bubble plot of KEGG enrichment analysis for upregulated and downregulated genes between fibroblasts from CON and WB muscle. (C) Pseudotime trajectory analysis of fibroblasts. (D) Representative images of Sirius Red staining in CON and WB muscle (scale bar = 50 µm, *n* = 8 birds per group). Collagen fibers are stained in red, while muscle fibers appear yellow. Quantification of the collagen‐positive area (%) is shown on the right. (E, F) Heatmap showing the dynamic changes of representative upregulated and downregulated genes in fibroblasts. (G) Dynamic changes in representative upregulated genes and fibroblast differentiation marker genes. Data are expressed as the mean ± SEM. Error bars represent the SEM of biological replicates. Statistical significance was assessed by unpaired two‐tailed Student's t‐test. ^*^ *p* < 0.05, ^**^ *p* < 0.01, ^***^ *p* < 0.001.

To better understand the fibrotic differentiation process, a pseudo‐developmental path was constructed using FBs from both groups (Figure 4C). Notably, FBs from the WB group were distributed at later stages of this trajectory, suggesting a more differentiated state along the inferred transcriptional continuum (Figure 4C). Consistently, Sirius Red staining demonstrated significantly higher collagen deposition in the WB group (Figure 4D). The expression patterns of DEGs were plotted as a function of pseudotime (Figure 4E,F). Most upregulated genes mapped toward the later end of this pseudotime ordering (Figure 4E), whereas the majority of downregulated genes mapped toward the initial end (Figure 4F). Among them, the characteristic genes for myofibroblast transition, including *FN1*, *COL1A1*, and *COL1A2*, showed concordant increases along this transcriptional ordering. Furthermore, among the top 5 upregulated TFs (Figure 4E), only *RUNX1* showed a continuously increasing expression pattern (Figure 4G). Therefore, the above data confirm fibrotic differentiation of FBs induced by WB and suggest *RUNX1* as an important regulator.

### 3.5 RUNX1 Promotes Activation and Proliferation While Inhibiting Differentiation in Isolated Primary Satellite Cells

The regulatory function of RUNX1 in the myogenic progression of SCs was investigated using siRNAs and overexpression plasmids in isolated SCs (Figure 5A–N). For knockdown experiments, the protein expression of RUNX1 was most markedly decreased by si‐577 among the three designed siRNAs; thus, si‐577 was selected for all subsequent functional experiments (labeled as si‐RUNX1) (Figure 5A). Knockdown of RUNX1 suppressed the expression of *PCNA* and *Cyclin D1*, suggesting reduced activation and proliferation of SCs (Figure 5B). Consistently, EdU incorporation assays further confirmed that RUNX1 knockdown significantly decreased the proportion of EdU‐positive SCs, indicating impaired proliferative capacity following RUNX1 depletion (Figure 5C). In agreement with these findings, the CCK‐8 assay showed that RUNX1 knockdown significantly reduced SC viability 24 h after transfection (Figure 5D). In isolated experiments for myogenic induction, immunofluorescence staining of MyHC showed a significantly higher myotube fusion index in the si‐RUNX1 group (Figure 5G). Consistently, the protein expression of MYOG was significantly enhanced, while no differences were found for MYOD (Figure 5E). The gene expression of *MYOG* was also dramatically increased, along with enhanced expression of *MyHC* (Figure 5F). In contrast, overexpression of RUNX1 (pcDNA3.1‐RUNX1) promoted the expression of *PCNA*, *CDK2*, and *Cyclin D1* (Figure 5I), and EdU incorporation assays further demonstrated that RUNX1 overexpression significantly increased the proportion of EdU‐positive SCs, supporting the role of RUNX1 in promoting SC proliferation (Figure 5J). Consistently, CCK‐8 analysis revealed that RUNX1 overexpression significantly increased SC viability 24 h after transfection (Figure 5K). However, overexpression of RUNX1 reduced the myotube fusion index (Figure 5N). In addition, both the protein expression of MYOD and MYOG and the gene expression of *MyHC* were remarkably decreased after overexpression of RUNX1 (Figure 5L,M). In summary, knockdown of RUNX1 inhibits proliferation but promotes differentiation of SCs, and vice versa.

**FIGURE 5:**
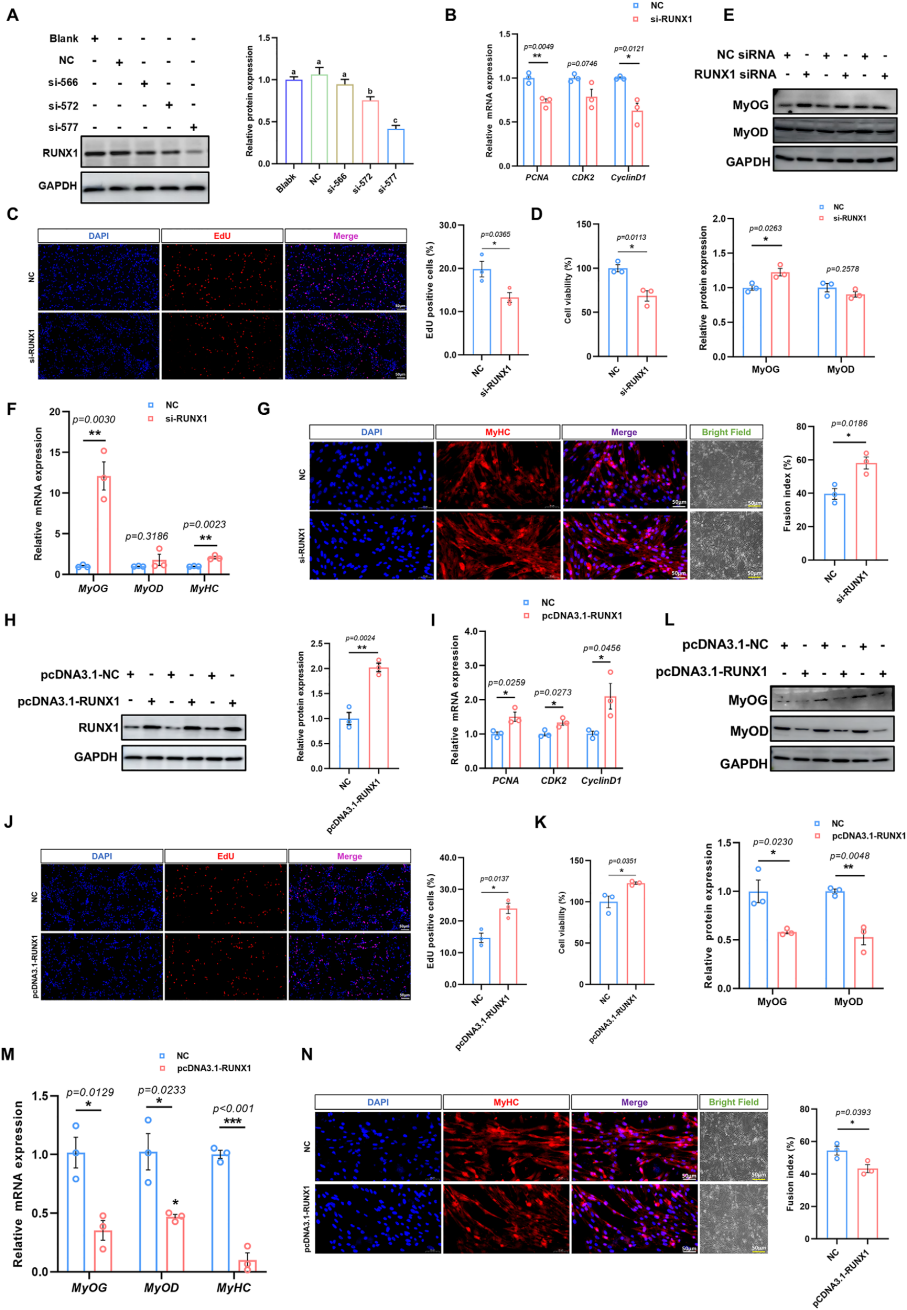
Effects of RUNX1 on satellite cell (SC) proliferation and differentiation. (A) Interference efficiency detection of the three designed siRNAs in SCs (*n* = 3). (B) Relative mRNA expression levels of proliferation‐related genes (*n* = 3). (C) Representative immunofluorescence images of EdU staining and quantitative analysis of EdU‐positive cells 24 h after RUNX1 knockdown in SCs (scale bar = 50 µm, *n* = 3). (D) CCK‐8 assay showing cell viability 24 h after RUNX1 knockdown in SCs (3 biological replicates, with 3 technical replicates per biological replicate). (E) Western blot analysis of MyOG and MyOD protein expression 48 h after induction of SC differentiation following RUNX1 knockdown (*n* = 3). (F) Relative mRNA expression levels of myogenic differentiation marker genes in SCs following RUNX1 knockdown (*n* = 3). (G) Representative immunofluorescence images and bright‐field images of MyHC (left) and quantitative analysis of the myotube fusion index (right) 48 h after inducing SCs differentiation (scale bar = 50 and 25 µm, *n* = 3). (H) Detection of RUNX1 overexpression efficiency in SCs (*n* = 3). (I) Relative mRNA expression levels of proliferation‐related genes (*n* = 3). (J) Representative immunofluorescence images of EdU staining and quantitative analysis of EdU‐positive cells 24 h after RUNX1 overexpression in SCs (scale bar = 50 µm, *n* = 3). (K) CCK‐8 assay showing cell viability 24 h after RUNX1 overexpression in SCs (3 biological replicates, with 3 technical replicates per biological replicate). (L) Western blot analysis of MyOG and MyOD protein expression 48 h after induction of SC differentiation following RUNX1 overexpression (*n* = 3). (M) Relative mRNA expression levels of myogenic differentiation marker genes in SCs following RUNX1 overexpression (*n* = 3). (N) Representative immunofluorescence images and bright‐field images of MyHC (left) and quantitative analysis of the myotube fusion index (right) 48 h after inducing SCs differentiation (scale bar = 50 and 25 µm, *n* = 3). Data are expressed as the mean ± SEM. Error bars represent the SEM of biological replicates. *n* = 3 represents three independent biological replicates, with each replicate generated by pooling SCs isolated from 2 to 3 embryos. For panels (C, G, J, N), each biological replicate was quantified from 3 random fields per condition. Statistical significance was assessed by one‐way ANOVA with Tukey's multiple comparisons (A) and unpaired two‐tailed Student's t‐test for two‐group comparisons (B–N). Different letters indicate differences between groups. ^*^ *p* < 0.05, ^**^ *p* < 0.01, ^***^ *p* < 0.001. GAPDH served as the housekeeping protein.

### 3.6 RUNX1 Promotes the Myofibroblast Transition of Chicken DF‐1 Fibroblasts

To assess the role of RUNX1 in fibroblast differentiation, we transfected chicken DF‐1 cells (an embryonic fibroblast cell line) with RUNX1 siRNA si‐577 (labeled as si‐RUNX1) or an overexpression plasmid, followed by stimulation with exogenous TGF‐β1 for 24 h. Transfection with si‐RUNX1 successfully reduced RUNX1 protein expression, as well as the protein levels of the myofibroblast markers α‐SMA and Collagen I (Figure 6B). Consistently, the fluorescence intensity of α‐SMA was significantly decreased in the si‐RUNX1 group (Figure 6A), along with the decreased gene expression of *RUNX1*, *ACTA2*, *FN1*, and *COL1A1* (Figure 6C). In contrast, RUNX1 overexpression significantly increased the protein expression levels of α‐SMA and Collagen I, the fluorescence intensity of α‐SMA, and the gene expression of *ACTA2*, *FN1*, and *COL1A1* (Figure 6D–F). Collectively, these gain‐ and loss‐of‐function experiments demonstrate that RUNX1 regulates the TGF‐β1‐induced myofibroblast transition of the chicken DF‐1 fibroblast model, supporting a candidate regulatory role in fibroblast fibrotic differentiation.

**FIGURE 6:**
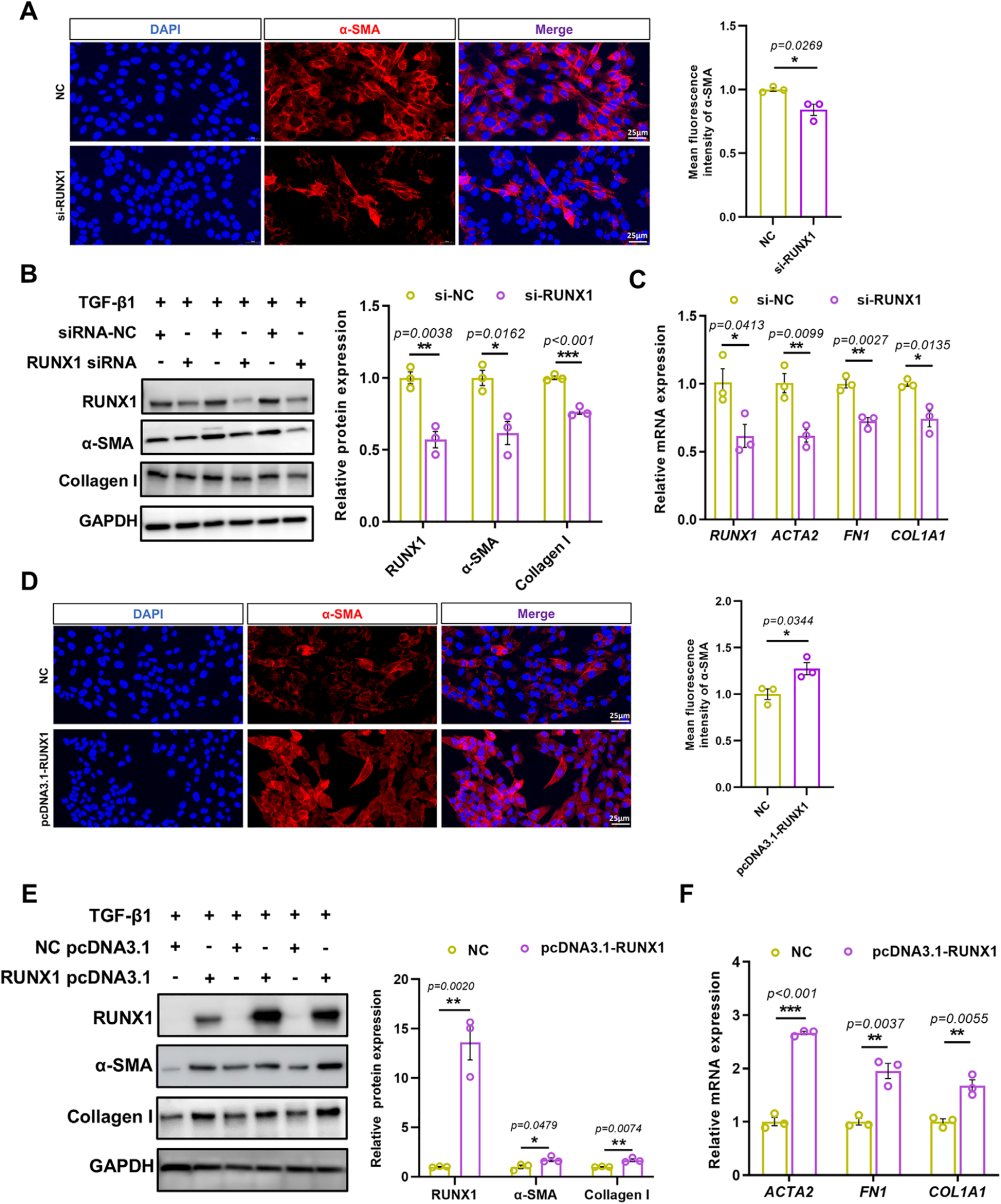
Effects of RUNX1 on fibroblast differentiation. (A–C) Effects of RUNX1 knockdown on fibroblasts. (A) Representative immunofluorescence images of α‐SMA staining (left) and quantification of mean fluorescence intensity of α‐SMA in RUNX1‐knockdown cells (right, scale bar = 25 µm, *n* = 3). (B) Representative Western blot analysis of RUNX1 and the myofibroblast‐associated proteins α‐SMA and Collagen I after TGF‐β1‐induced differentiation (*n* = 3). (C) Relative mRNA expression levels of differentiation marker genes (*n* = 3). (D–F) Effects of RUNX1 overexpression on fibroblasts. (D) Representative immunofluorescence images of α‐SMA staining (left) and quantification of mean fluorescence intensity of α‐SMA (right, scale bar = 25 µm, *n* = 3). (E) Representative Western blot analysis of RUNX1, α‐SMA, and Collagen I after TGF‐β1‐induced differentiation (*n* = 3). (F) Relative mRNA expression levels of differentiation marker genes (*n* = 3). For panels (A and D), each biological replicate was quantified from 3 random fields per condition. *n* = 3 independent biological replicates (independent batch); error bars represent the SEM of biological replicates. Data are expressed as the mean ± SEM. Statistical significance was assessed by an unpaired two‐tailed Student's t‐test for two‐group comparisons. ^*^ *p* < 0.05, ^**^ *p* < 0.01, ^***^ *p* < 0.001. GAPDH served as the housekeeping protein.

### 3.7 Hypoxia is Associated With Increased RUNX1 Expression in WB Myopathy

The above results showed enhanced expression of *RUNX1* in both SCs and FBs. Consistently, both the mRNA and protein expression levels of RUNX1 were enhanced in muscle samples of the WB group compared to those of the CON group (Figure 7A,B). Since the development of WB is associated with reduced vascular density, we evaluated the hypoxic state in the breast muscle by analysis of blood flow and gas concentrations. The results revealed that the blood flow (Figure 7C,D), pO_2_, and sO_2_ in WB were significantly lower than those in normal muscles (Figure 7E,F). Although wing‐vein blood gas reflects systemic rather than intramuscular oxygenation, the combination of reduced perfusion and lower systemic oxygenation might reflect a potential hypoxic state in WB muscle. Moreover, we investigated the relationship between hypoxia and RUNX1 expression in isolated SCs and DF‐1 FBs. Hypoxia was induced by incubating cells under 1% O_2_ conditions for 24 h. Enhanced protein levels of HIF‐1α in both SCs and FBs confirmed hypoxic induction in these cells (Figure 7G–J). Consistently, the expression of RUNX1 was also induced in both cell types (Figure 7G–J). Therefore, the potential hypoxic state in WB muscle was accompanied by increased RUNX1 expression in both SCs and FBs, consistent with our in vitro finding that severe hypoxia (1% O_2_) is sufficient to induce RUNX1 upregulation. Together with the enhanced activation and proliferation but impaired differentiation of SCs, and the excessive fibrotic differentiation of FBs, these changes might contribute to the pathological degeneration in WB myopathy.

**FIGURE 7:**
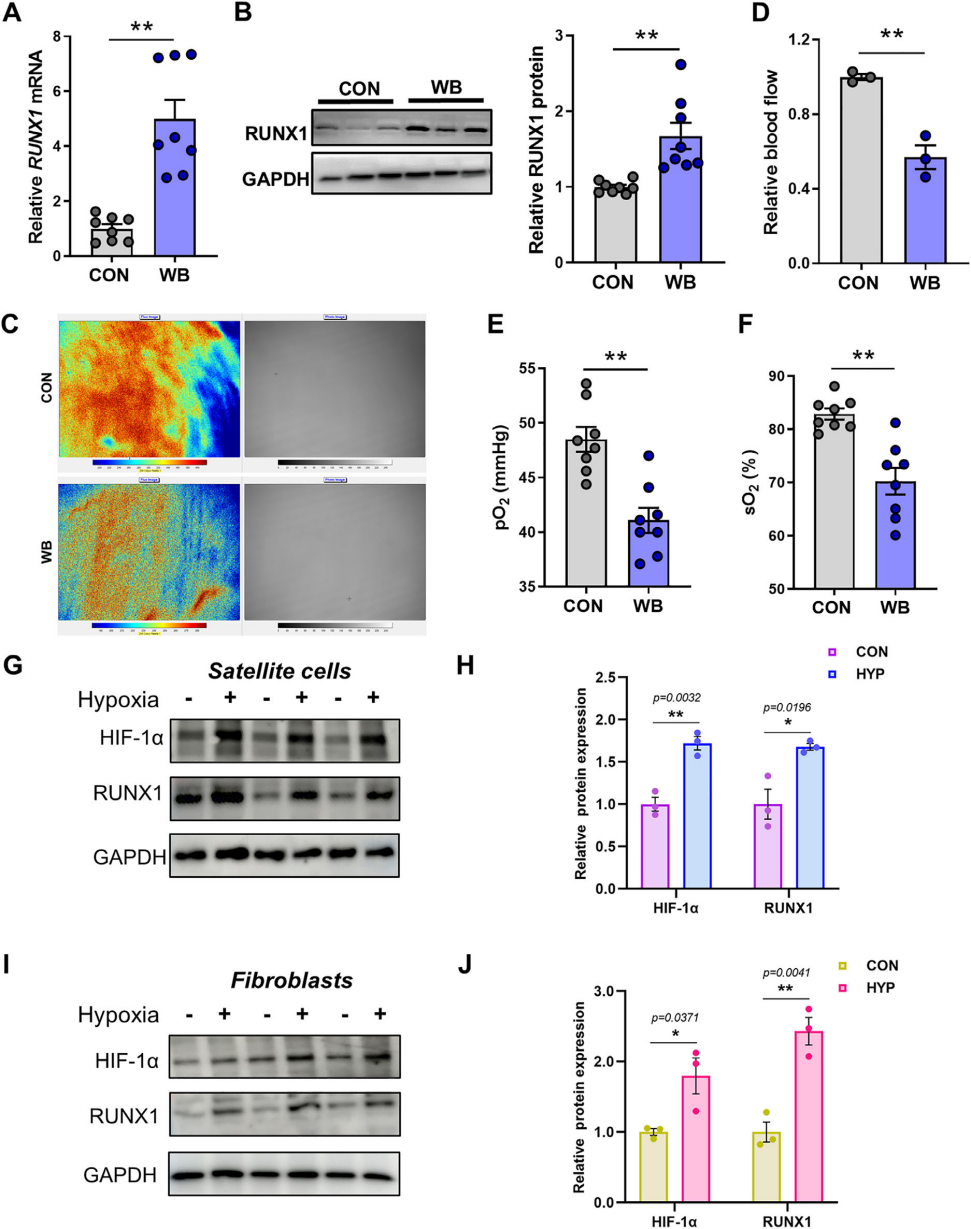
Association between hypoxia‐related indicators and RUNX1 expression in wooden breast (WB) muscle. (A) Relative expression levels of RUNX1 mRNA in CON and WB muscles (*n* = 8 birds per group). (B) Relative protein expression levels of RUNX1 in CON and WB muscles (*n* = 8 birds per group). (C, D) Representative laser speckle flow imaging and quantitative histograms of blood flow results in CON and WB muscles (*n* = 3 birds per group). (E, F) Comparative analysis of oxygen partial pressure (pO2) and oxygen saturation (sO2) in wing‐vein blood gas measurements from CON and WB broilers (*n* = 8 birds per group). (G, H) Relative protein expression levels of HIF‐1α and RUNX1 in satellite cells 24 h after hypoxia induction (*n* = 3). *n* = 3 represents three independent biological replicates, with each replicate generated by pooling SCs isolated from 2 to 3 embryos. (I, J) Relative protein expression levels of HIF‐1α and RUNX1 in fibroblasts 24 h after hypoxia induction (*n* = 3). *n* = 3 independent biological replicates (independent batches). Error bars represent the SEM of biological replicates. Data are expressed as the mean ± SEM. Statistical significance was assessed by an unpaired two‐tailed Student's t‐test for two‐group comparisons. ^*^ *p* < 0.05, ^**^ *p* < 0.01, ^***^ *p* < 0.001. GAPDH is a housekeeping protein.

### 3.8 Wooden Breast Myopathy Disrupts Intercellular Signaling Communications Contributing to Pathological Degeneration

Changes in the composition and function of breast muscle cells caused by WB would disrupt the intercellular signaling networks required for efficient regeneration. Therefore, we investigated cell‐cell communication changes using the R package CellChat. A substantial increase in the number of intercellular interactions was found in the WB group, while the changes in interaction strength were minimal (Figure 8A,B). The main changes in signal sources originated from the Myo1, Fibro, SC, and Endo clusters, whereas signals received by Myo1, SC, and Endo clusters were the most reduced (Figure 8A). By quantitatively analyzing the outgoing and incoming interaction strengths to identify major signaling sources and targets, we found that the Fibro cluster (FBs) exhibited the strongest capacity for both signal release and reception in the breast muscle compared to other cell groups (Figure 8C). Moreover, the outgoing signaling strength of Fibro was further enhanced in the WB group. Meanwhile, both outgoing and incoming interaction strengths of SCs were more pronounced in the WB group (Figure 8C). Additionally, both the outgoing and incoming interaction strengths of Endo were decreased, possibly due to reduced vascularization caused by WB. Furthermore, we analyzed the ligand‐receptor pairs between the cell clusters identified above that showed the most changes as sources and receivers (Figure 8D). Notably, TGFB signaling pairs, secreted by Fibro, SCs, and Endo, and received by Myo1 and SCs, were consistently enhanced in the WB group. In addition, MDK signaling pairs, secreted by Fibro and received by other cell types, were also consistently enhanced. To further validate the enhanced TGFB signaling predicted by CellChat, we examined the expression of TGFB ligands in fibroblasts and their corresponding receptors in SCs. Hypoxia treatment for 24 h significantly increased the mRNA expression of *TGFB1* and *TGFB3* in fibroblasts (Figure 8E). Consistently, treatment of SCs with conditioned medium from hypoxia‐treated fibroblasts for 24 h significantly increased the mRNA expression of the TGFB receptors *TGFBR1* and *TGFBR2* (Figure 8F). Therefore, these observations are consistent with a possible contribution of enhanced TGFB and MDK signaling to the impaired myogenic progression of SCs and myofiber degeneration in the WB group.

**FIGURE 8:**
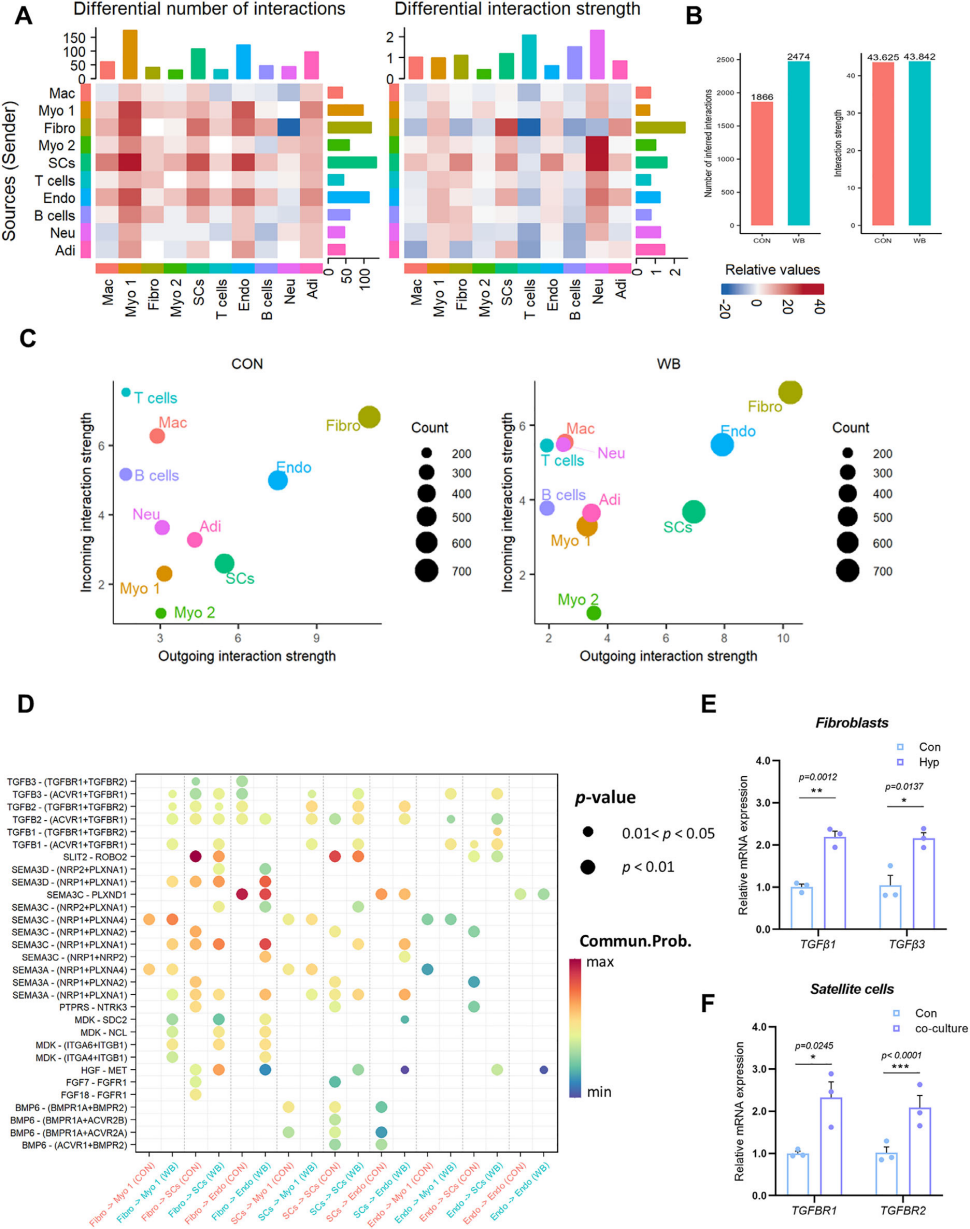
Comparison of cell communication between normal and wooden breast (WB) muscle. (A, B) The number and strength of inferred communication links between normal and WB muscle. (C) Differential interaction analysis identifying prominently altered signaling sources and targets in CON and WB muscle. x‐axis and y‐axis are the total outgoing or incoming communication probability associated with each cell cluster. (D) Comparisons of significant interactions between ligand‐receptor pairs between CON and WB muscle. Dot color reflects communication probabilities, and dot size represents computed p‐values. Empty space means the communication probability is zero. p‐values are computed from a one‐sided permutation test. (E) Relative mRNA expression levels of *TGFβ1* and *TGFβ3* in fibroblasts after 24 h of hypoxia treatment (*n* = 3). *n* = 3 independent biological replicates (independent batches). (F) Relative mRNA expression levels of *TGFBR1* and *TGFBR2* in satellite cells after 24 h of treatment with conditioned medium collected from hypoxia‐treated fibroblasts (*n* = 3). *n* = 3 represents three independent biological replicates, with each replicate generated by pooling SCs isolated from 2 to 3 embryos. Data are expressed as the mean ± SEM. Error bars represent the SEM of biological replicates. Statistical significance was assessed by an unpaired two‐tailed Student's t‐test for two‐group comparisons. ^*^ *p* < 0.05, ^**^ *p* < 0.01, ^***^ *p* < 0.001.

### 3.9 Consistent Expression Pattern of RUNX1 in Human and Mouse Muscles

Publicly available muscle datasets from humans and mice were re‐analyzed to reveal the expression patterns of *RUNX1*. First, we evaluated the expression pattern of *RUNX* family members (*RUNX*1‐3) and *HIF1A* in different cell types of human skeletal muscle under physiological conditions (Figure 9A). This snRNA‐seq dataset includes samples from 10 different muscle regions of humans and annotates 16 cell types, including immune cells, SCs, endothelial cells, myonuclei, FBs, and adipocytes (https://cells.ucsc.edu/?ds=muscle‐cell‐atlas) [37]. Consistent with the above findings in broiler muscle, *RUNX1* was found to be highly expressed in both differentiating fibroblasts (*COL1A1* ^+^) and activated SCs (P*AX7* ^−^/*MYF5* ^+^, Figure 9A), which was also accompanied by relatively higher expression of *HIF1A*. However, the expression of *RUNX2* and *RUNX3* was barely detectable in skeletal muscle.

**FIGURE 9:**
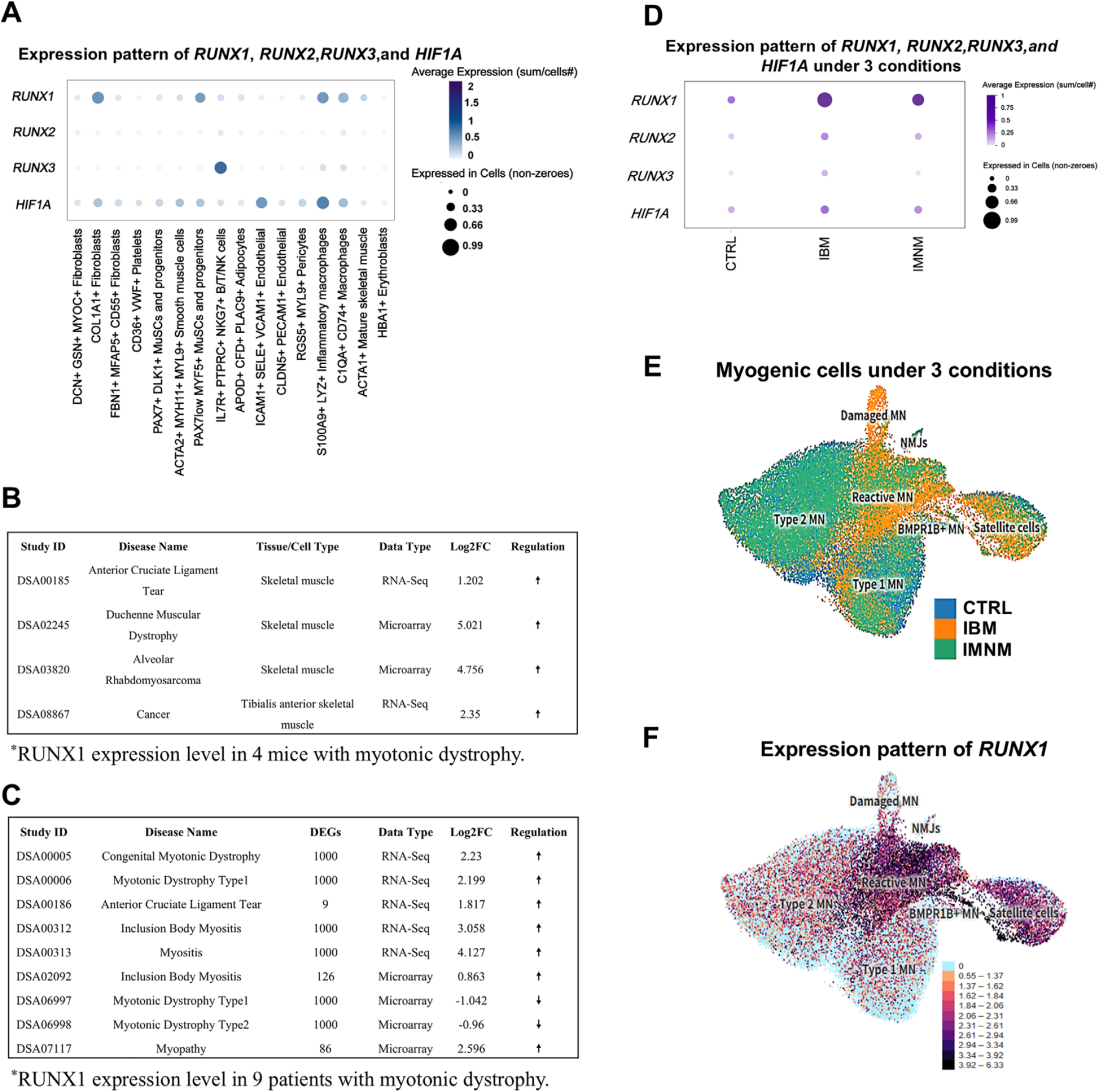
Integrated analysis of human and mouse skeletal muscle datasets. For (A–D), snRNA‐seq datasets for human and mouse skeletal muscle cells were obtained and modified from the interactive tools of the UCSC Cell Browser (https://cells.ucsc.edu/) and the CNSknowall website (https://www.cnsknowall.com/). (A) Dot plot showing the expression levels of the three RUNX family members (*RUNX1*, *RUNX2*, and *RUNX3*) and *HIF1A* across the annotated cell clusters. (B, C) Summary of *RUNX1* expression in skeletal muscle from mouse models of muscle disease (B) and human patients with various muscle diseases (C), based on transcriptome sequencing and microarray datasets obtained from the DiSignAtlas (http://www.inbirg.com/disignatlas). (D) Dot plot showing the expression pattern of *RUNX1* and *HIF1A* under 3 conditions (CTRL, IBM, and IMNM). (E) UMAP showing the distribution of myogenic cells under the 3 conditions. (F) Feature plot showing the expression pattern of *RUNX1* mapped onto the UMAP. IBM: Inclusion Body Myositis; IMNM: Immune‐Mediated Necrotizing Myopathy.

Furthermore, we checked *RUNX1* expression levels in skeletal muscle samples from nine patients with myotonic dystrophy and four mice with myotonic dystrophy (Figure 9B,C) using the transcriptome sequencing or microarray data analyzed and presented by the DiSignAtlas database (http://www.inbirg.com/disignatlas). We found that *RUNX1* was downregulated only in the skeletal muscle datasets of two patients with myotonic dystrophy, while its expression level was upregulated in the remaining datasets. In addition, we compared the expression levels of *RUNX1* in skeletal muscle tissue from datasets of 9 patients with myopathy (https://cells.ucsc.edu/?ds=muscle‐ibm+myonuclei) [38]. Consistent with our findings, the expression levels of *RUNX1* were higher in patients with Inclusion Body Myositis (IBM) and Immune‐Mediated Necrotizing Myopathy (IMNM, Figure 9D), which were also accompanied by relatively higher expression of *HIF1A*. The expression of *RUNX2* and *RUNX3* was also barely detectable in these datasets of myopathies. Notably, the expression pattern of *RUNX1* mirrors the distribution of cells in IBM, suggesting the important regulatory function of RUNX1 in the development of IBM (Figure 9E,F). Overall, this finding suggests a conserved function of HIF1A and RUNX1 in the development of muscle diseases or disorders.

## 4 Discussion

Skeletal muscle serves important functions in physical support and metabolic health in humans and animals. Loss of muscle mass and function resulting from compromised myogenic repair after injury under diseased or disordered conditions can directly disrupt whole‐body metabolic homeostasis, threatening human quality of life and animal production efficiency [40]. In this study, broiler WB myopathy was used as a model to investigate the pathological origins of myofiber degeneration and excessive fibrosis in skeletal muscle. Combined analysis of snRNA‐seq, in vivo and in vitro studies identified hypoxia‐associated RUNX1 upregulation as a major regulator of the disrupted myogenic progression of SCs, the excessive fibrotic differentiation of FBs, and disruption of the local signaling niche required for proper muscle repair. Because this cross‐sectional comparison was based on eight histologically confirmed normal and eight severe WB muscles selected as extreme phenotypes rather than a representative disease spectrum, and because the snRNA‐seq libraries were generated from pooled tissues, the sequencing‐derived results alone do not establish temporal disease progression; nevertheless, the key patterns were corroborated by independent biological replicates and by functional cell‐culture experiments, together supporting a contribution of RUNX1 to WB pathology.

Muscle growth after birth primarily results from the hypertrophic growth of myofibers [41]. Significant increases in myofiber diameter of those fast‐growing breeds reduce the available space for vasculature, leading to an overall decrease in total blood vessels [15, 23, 42]. Insufficient vascularization may impair the supply of nutrients and O_2_ to the breast muscle, while also hindering the removal of CO_2_ and metabolic waste products, ultimately triggering oxidative stress and metabolic disturbances [43, 44]. Consistently, results of this study showed that the relative blood flow in the breast muscle of WB‐affected broilers was significantly lower than that in normal breast muscle. Similarly, our blood gas analysis revealed significantly lower pO_2_ and sO_2_, and significantly higher pCO_2_ in WB‐affected broilers. Because laser speckle imaging reports local perfusion and wing‐vein blood gas reflects systemic rather than intramuscular oxygenation, these measurements do not directly quantify the oxygen tension within the pectoralis major; nevertheless, the combination of reduced perfusion and lower systemic oxygenation may be consistent with a potential hypoxic state in WB muscle. In addition, studies have shown that hypoxia leads to oxidative stress and inflammation in injured muscle [45, 46]. Our snRNA‐seq transcriptomic analysis of myofibers, although based on one pooled library per condition, revealed enhanced oxidative stress and inflammation in the WB group and increased remodeling of myofibers toward these pathological features, which were independently corroborated by the ultrastructural observations described above. Moreover, increased ROS production under hypoxic conditions leads to mitochondrial dysfunction, a process increasingly recognized as a conserved pathological driver and therapeutic target in muscle and cardiac myopathies [43, 47, 48]. Our ultrastructural observation clearly revealed mitochondrial vacuolization and swelling in the WB muscle. Mitochondrial dysfunction in WB muscle would reduce energy supply to myofibers by decreasing oxidative phosphorylation and diminishing ATP production, ultimately resulting in myofibrillar necrosis and dissolution as shown by our ultrastructural observations [49, 50]. Additionally, those stressed myofibers in WB increased secretion of multiple key profibrotic cytokines (e.g., *DCN*, *TGFB3*, *MMP9*, *MMP1*), which may further exacerbate the fibrotic differentiation of FBs.

Muscle regeneration efficiency is determined by the myogenic contribution of SCs. Upon injury, SCs exit quiescence, then proliferate, differentiate, and fuse to form new myofibers or repair existing ones. This myogenic process is well orchestrated by both intrinsic and extrinsic signals from the skeletal muscle niche, and impaired niche‐driven myogenesis is a shared feature of metabolic myopathies that limits muscle repair [51, 52]. Chronic hypoxia has been shown to result in muscle atrophy and weakness in mice, along with increased fibrosis [24]. The transcription factors HIF‐1α and HIF‐2α were found to play distinct roles under chronic hypoxia. While HIF‐2α ablation in mice rescued the activation and proliferation defects under hypoxia, HIF‐1α ablation resulted in reduced activation and proliferation of SCs [24]. Furthermore, double knockout of HIF‐1α and HIF‐2α in mouse myoblasts reduced self‐renewal but promoted differentiation under hypoxic conditions by regulating Notch signaling [53]. In addition, studies also showed that severe hypoxia (≤ 1% O_2_) was detrimental for myogenic differentiation in vitro, whereas a 3%–6% O_2_ level could promote myogenesis [54]. Consistently, our results suggested a potential hypoxic state in the WB muscle, as indicated by enhanced HIF‐1α expression together with reduced blood flow and oxygen supply. Moreover, we observed a strong positive correlation between HIF‐1α and RUNX1 expression in WB muscle. In cultured SCs, functional experiments in which RUNX1 was knocked down or overexpressed showed that elevated RUNX1 promoted proliferation while suppressing differentiation. These knockdown findings were based on a single siRNA, and because no rescue experiment was performed, sequence‐specific or transfection‐related effects cannot be fully excluded; nevertheless, the concordant reciprocal results of overexpression support a specific role for RUNX1. Therefore, hypoxia‐related signaling and HIF‐1α upregulation may be closely associated with the disrupted myogenic progression of SCs and myofiber necrosis, which occur alongside RUNX1 upregulation. Consistent with our findings, hypoxia is increasingly recognized as a context‐dependent regulator of tissue remodeling. During acute repair, transient hypoxia activates pathways associated with cellular migration and regeneration, whereas persistent hypoxia promotes pathological remodeling and fibrosis. For instance, short‐term hypoxia in wound healing has been shown to enhance epithelial migration via the CCL2–ERK1/2 pathway and epithelial–mesenchymal transition, underscoring the reparative function of early hypoxic responses. Conversely, chronic hypoxia is linked to exacerbated fibrotic remodeling through the sustained activation of HIF‐dependent signaling. In conjunction with our results, these observations suggest that the duration and cellular context of hypoxia may dictate whether hypoxic signaling facilitates tissue regeneration or drives pathological fibrosis [25].

The myogenic capacity of SCs is also affected by other cell types within the skeletal muscle niche, especially FBs. At the initial stage of injury, FBs are activated and undergo myofibroblast transition to produce ECM, forming a temporary scaffold that supports the survival, migration, and differentiation of SCs [55, 56]. Moreover, FBs regulate the myogenic capacity of SCs by secreting signals including WISP1, FGF7, IGF1, IL6, IL10, WNTs, and follistatin under healthy conditions [16, 57, 58]. This myogenic‐promoting process is transient, and FBs undergo apoptosis or return to quiescence after efficient repair. Pathological conditions disrupt this dynamic transition of activation and quiescence, leading to excessive fibrosis. Previous studies in our laboratory have also demonstrated the continuous activation and differentiation of FBs with excessive production of ECM, especially collagen I and fibronectin in the WB muscle [15]. In this study, our loss‐ and gain‐of‐function experiments suggested that RUNX1 regulates the fibrotic differentiation of FBs: knockdown of RUNX1 in the chicken DF‐1 cell line reduced their myofibroblast transition, whereas RUNX1 overexpression had the opposite effect. However, DF‐1 is an embryonic chicken fibroblast cell line and was examined under exogenous TGF‐β1 stimulation, whereas the disease‐relevant fibroblasts identified by our snRNA‐seq are adult muscle‐resident fibroblasts (potentially including fibro‐adipogenic progenitors). The DF‐1 experiments therefore provide proof‐of‐principle evidence that RUNX1 can modulate fibrotic‐marker expression in a tractable chicken fibroblast system, but they do not by themselves establish that the identical response occurs in adult WB fibroblasts or fibro‐adipogenic progenitors. Furthermore, hypoxia has been demonstrated to activate the TGF‐β1/SMAD signaling pathway via HIF‐1α signaling, leading to enhanced collagen deposition in human dermal fibroblasts [59]. Consistent with this, in our system the profibrotic effects accompanying hypoxia and HIF‐1α upregulation occurred concurrently with RUNX1 upregulation, although a causative relationship cannot be established from these in vivo data. Additionally, CellChat analysis of the pooled libraries indicated that FBs are the main signaling sources within broiler muscle, with noticeably enhanced TGFB and MDK signaling pairs between FBs and SCs and between FBs and myofibers. The predicted TGFB signaling was further supported by targeted in vitro experiments, collectively suggesting an anti‐myogenic role of FBs in WB muscle. While our current findings highlight the collective impact of FBs, recent mammalian atlases have revealed extensive fibroblast heterogeneity with distinct spatial and functional subtypes [60, 61, 62]. Because these functional experiments were performed in the embryonic DF‐1 cell line, they cannot resolve whether RUNX1 acts equivalently across these adult fibroblast/Fibro‐adipogenic progenitor subpopulations. Currently, exploring such specific subsets in avian models is limited by the lack of consensus classifications and species‐specific antibodies. Therefore, defining the fine‐scale subpopulations of chicken fibroblasts and their precise roles in the myopathic microenvironment, as well as validating RUNX1 function in adult muscle‐resident fibroblasts, represents a critical avenue for future research.

RUNX1 is an evolutionarily conserved transcription factor involved in diverse biological processes such as hematopoiesis, immune regulation, and adipocyte thermogenesis [63, 64, 65]. It was also proposed as a potential therapeutic target for the treatment of cardiovascular diseases due to its indispensable role in definitive hematopoiesis [64]. Similarly, we also reported that elevated RUNX1 expression was associated with vascular degeneration and hypoxia in the WB muscle. Based on these findings, we hypothesize that targeting RUNX1 may facilitate muscle regeneration through the regulation of SCs and FBs and may attenuate fibrotic remodeling; whether such intervention would also improve muscle perfusion and oxygenation remains to be tested in future in vivo studies.

## 5 Conclusion

In this study, WB myopathy in broilers was used as a model to investigate the pathological origins of the co‐occurrence of myofiber degeneration and fibrosis. The study revealed that pronounced myofiber hypertrophy was associated with diminished vascular density and enhanced hypoxia‐responsive signaling in WB‑affected muscle. This potentially hypoxic environment was accompanied by increased RUNX1 expression in breast muscle, and functional experiments indicated that elevated RUNX1 stimulated SC activation and proliferation while inhibiting their differentiation. Additionally, in a chicken fibroblast cell‐culture model, RUNX1 promoted the myofibroblast transition of fibroblasts and increased their secretion of anti‐myogenic factors, a finding that requires further validation in adult muscle‐resident fibroblasts. The resultant disruption of SC myogenic progression, together with the disruption of the myogenic niche, facilitated myofiber necrosis and structural deterioration. Comparative transcriptomic analyses of human and mouse myopathies demonstrated that the co‐expression pattern of HIF‐1A and RUNX1 is highly conserved among species. Overall, these findings elucidate the pivotal role of RUNX1 in muscle pathology and highlight it as a candidate for future in vivo therapeutic targeting studies.

## Author Contributions

L.Z. and T.H. contributed equally to this work. L.Z.: writing – review & editing, methodology, and investigation. T.H.: writing – original draft, methodology, and investigation. L.Z.: reviewing & editing. F.G.: review & editing, and investigation. T.X.: writing – review & editing, supervision, resources, project administration, funding acquisition, and conceptualization.

## Funding

This work was funded by the National Natural Science Foundation of China (32272900), the National Key Research and Development Program of China (2023YFD1301304), the Fundamental Research Funds for the Central Universities (YDZX2026007), the Natural Science Foundation of Jiangsu Province (BK20252059), and the National Natural Science Foundation of China (32573161).

## Conflicts of Interest

The authors declare no conflicts of interest.

## AI Disclosure

The authors declare that no artificial intelligence (AI) tools or AI‐assisted technologies were used in the preparation, writing, or editing of this manuscript.

## Supporting information




**Supporting File 1**: advs78533‐sup‐0001‐SuppMat.docx.

**Supporting File 2**: advs78533‐sup‐0002‐Tables.docx.

**Supporting File 3**: advs78533‐sup‐0003‐SuppMat.pdf.

## Data Availability

The data that support the findings of this study are available from the corresponding author upon reasonable request. The raw sequence data from this study have been deposited in the Genome Sequence Archive (Genomics, Proteomics & Bioinformatics 2021) in National Genomics Data Center (Nucleic Acids Res 2022), China National Center for Bioinformation / Beijing Institute of Genomics, Chinese Academy of Sciences (GSA: CRA034454) that are publicly accessible at https://ngdc.cncb.ac.cn/gsa.
