# Supplementary material for: RUNX1‐Mediated Hypoxic Signaling Impairs Myogenesis and Promotes Fibrosis in Myopathies
Source: Adv Sci (Weinh). 2026 Oct 31:e78533. Online ahead of print. doi: 10.1002/advs.78533 (PMC13723716; doi:10.1002/advs.78533)
Supplement: Supplementary file 1 [file ADVS-9999-e78533-s001.pdf]

Supplementary File S3. Visual mapping of real-time blood flow in the cranial region of the broiler pectoralis major muscle.

CON1

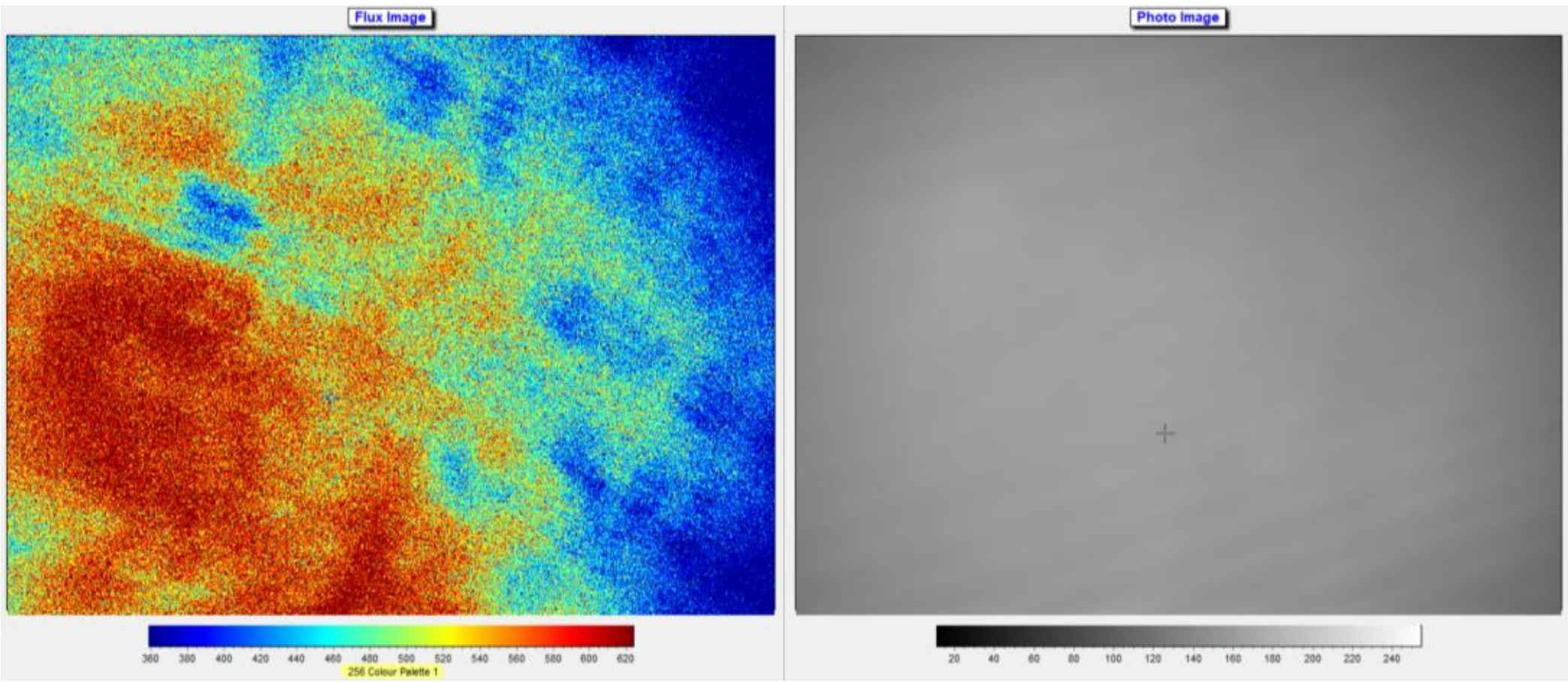

CON2

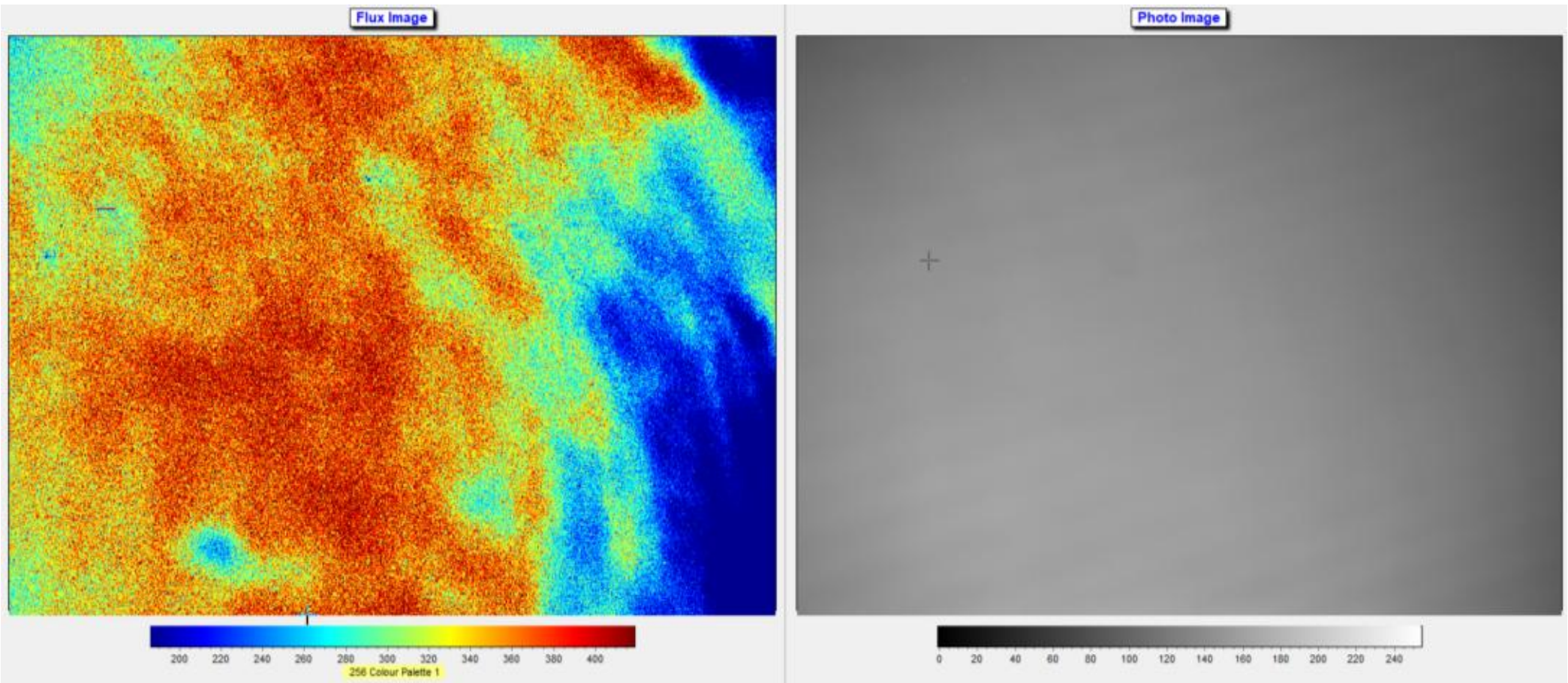

CON3

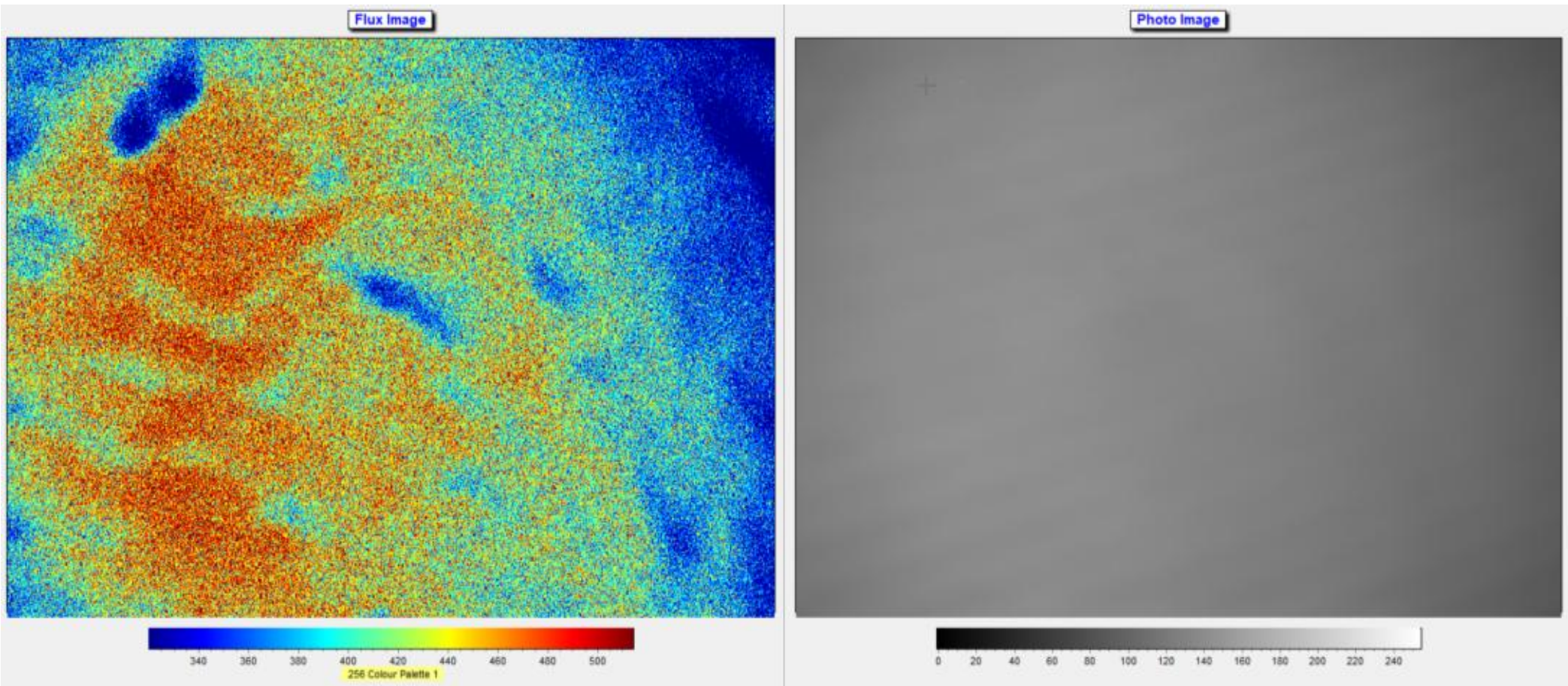

WB1

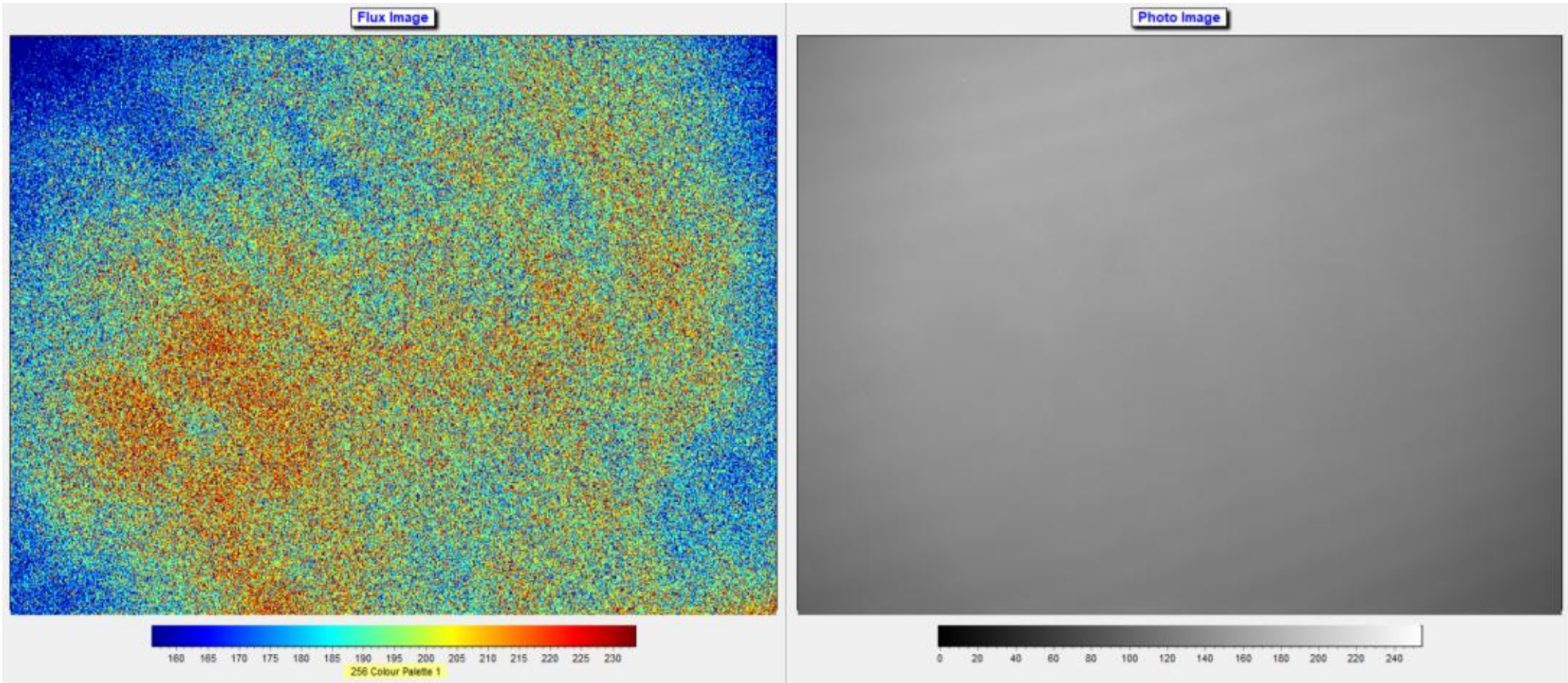
Flux Image
160 165 170 175 180 185 190 195 200 205 210 215 220 225 230
256 Colour Palette 1
Photo Image
0 20 40 60 80 100 120 140 160 180 200 220 240

WB2

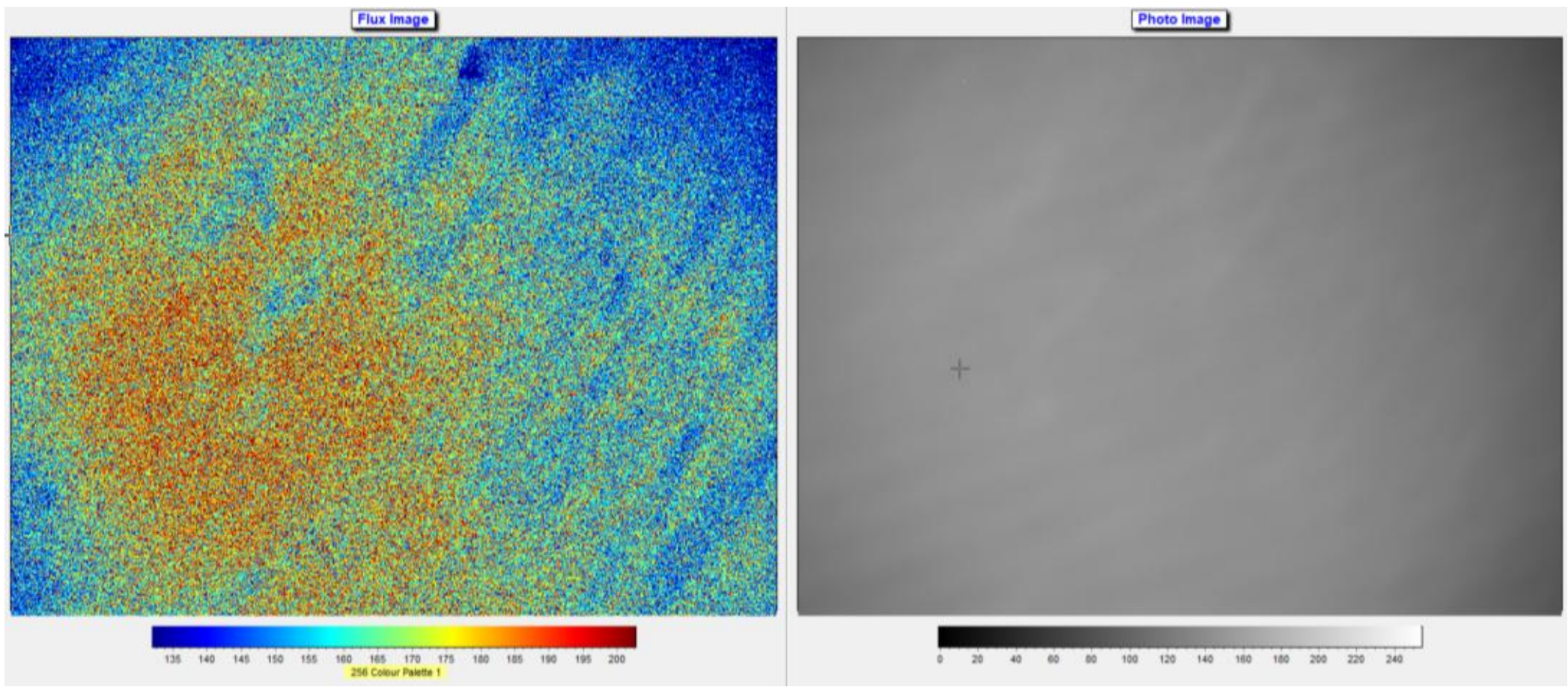
Flux Image
135 140 145 150 155 160 165 170 175 180 185 190 195 200
256 Colour Palette 1
Photo Image
0 20 40 60 80 100 120 140 160 180 200 220 240

WB3

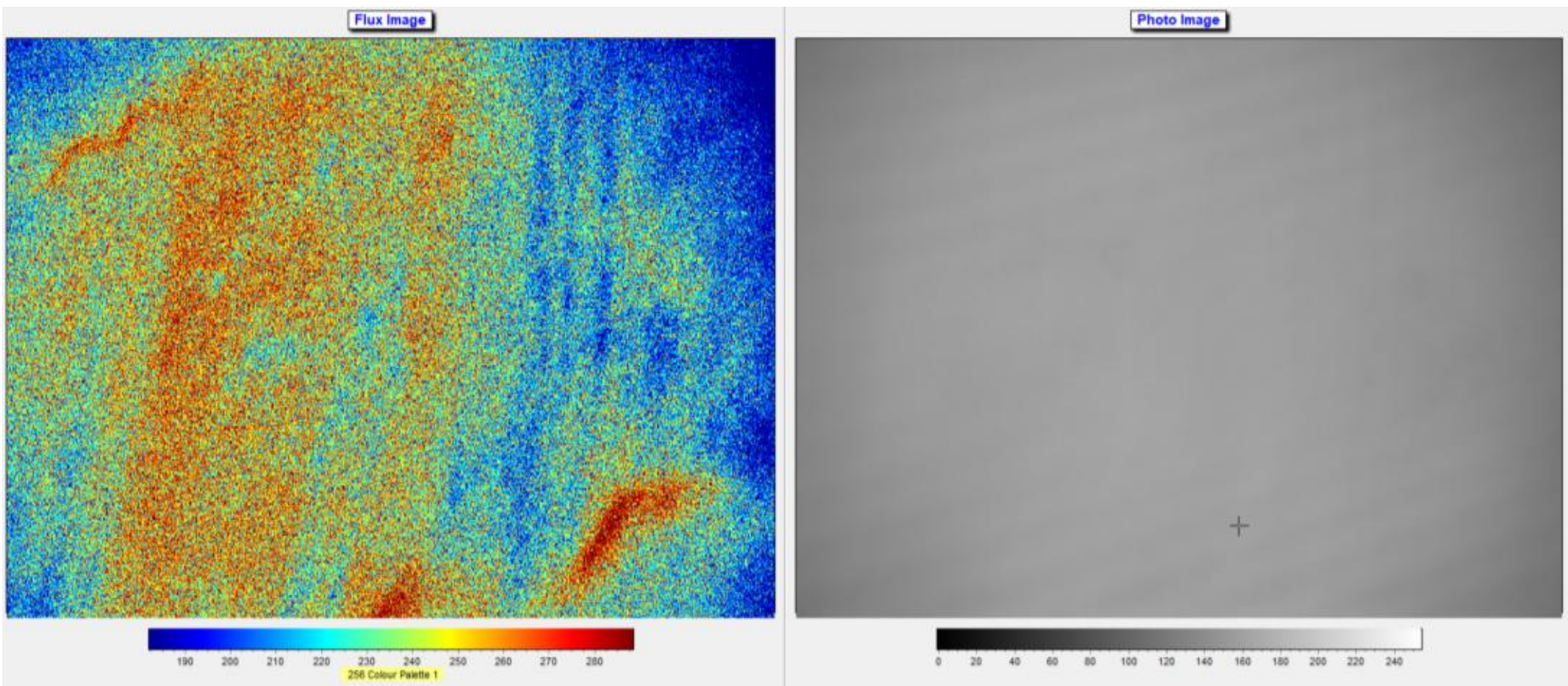
Flux Image
190 200 210 220 230 240 250 260 270 280
256 Colour Palette 1
Photo Image
0 20 40 60 80 100 120 140 160 180 200 220 240
