# Supplementary material for: RUNX1‐Mediated Hypoxic Signaling Impairs Myogenesis and Promotes Fibrosis in Myopathies
Source: Adv Sci (Weinh). 2026 Oct 31:e78533. Online ahead of print. doi: 10.1002/advs.78533 (PMC13723716; doi:10.1002/advs.78533)
Supplement: Supplementary file 2 [file ADVS-9999-e78533-s002.docx]

**RUNX1-Mediated Hypoxic Signaling Impairs Myogenesis and Promotes Fibrosis in Myopathies**

*Liang Zhao, Taijiang Hou, Lin Zhang, Feng Gao, and Tong Xing**

L. Zhao, T. Hou, L. Zhang, F. Gao

College of Animal Science and Technology, Nanjing Agricultural University

Nanjing, Jiangsu 210095, China

Email: liang.zhao@njau.edu.cn; 15083105622@163.com

ORCID: https://orcid.org/0000-0003-2446-5577

T. Xing

College of Animal Science and Technology, State Key Laboratory of Meat Quality Control and Cultured Meat Development, Nanjing Agricultural University

Nanjing, Jiangsu 210095, China

E-mail: xingtong@njau.edu.cn

ORCID: https://orcid.org/0000-0003-1561-4614

Funding: National Natural Science Foundation of China (32272900), the National Key Research and Development Program of China (2023YFD1301304), Fundamental Research Funds for the Central Universities (YDZX2026007) and Natural Science Foundation of Jiangsu Province (BK20252059).

Keywords: Myopathy; Myogenic Repair; Fibrosis; Hypoxia; RUNX1

**1. Satellite cell culture**

Primary satellite cells were isolated from the PM muscles of 12-day-old AA broiler embryos. Embryonated eggs were sprayed with 75% ethanol and placed in a biosafety cabinet. Embryos were aseptically removed using forceps and transferred to a petri dish containing pre-warmed PBS. The PM muscles were dissected, bones and residual connective tissue were removed, and muscles were rinsed in PBS before transfer to a watch glass. Muscles were minced into a paste-like consistency using scissors and digested with 1-2 mL of trypsin until the tissue fragmented into fine particles. Digestion was immediately terminated by adding 3 volumes of complete DMEM/F12 medium (supplemented with 10% FBS and 1% penicillin/streptomycin). The resulting suspension was sequentially filtered through 200-mesh and 100-mesh cell strainers. The filtrate was collected and centrifuged at 1000 rpm for 5 minutes. The pellet was resuspended and subjected to differential adhesion for 45 minutes at 37°C under 5% CO₂. Non-adherent cells were then transferred to culture flasks and maintained at 37°C with 5% CO₂. Cells were used for subsequent experiments after the first passage.

Following isolation and one passage, primary satellite cells were proliferated in complete DMEM/F12 medium (11320033, Gibco, Grand Island, NE, USA) for 24 h. Upon reaching 80% confluence, the medium was replaced with differentiation medium (97% DMEM/F12, 2% pregnant mare serum, 1% penicillin/streptomycin) to induce differentiation for 48 h.

**2. Fibroblast cell culture**

The chicken embryo fibroblast cell line DF-1 was purchased from Wuhan Sunncell Bio-Technology Co., Ltd. (SNL-006, Wuhan, China). Cells were maintained in DMEM medium (Gibco, Grand Island, NE, USA) supplemented with 10% FBS (BS-1105, Opcell, Inner Mongolia Autonomous Region, China) and 1% penicillin/streptomycin (PB180120. Procell, Wuhan, China), under a humidified atmosphere of 5% CO₂ at 37°C. Upon reaching confluence, cells were detached using 0.25 % trypsin-EDTA solution (25200056, Gibco, Grand Island, NE, USA) and passaged into new culture dishes. After reaching a stable growth status, cells were treated with 10 ng/mL TGF-β1 (HY-P7118, MedChemExpress, USA) for 24 h to induce differentiation.
