# Supplementary material for: RUNX1‐Mediated Hypoxic Signaling Impairs Myogenesis and Promotes Fibrosis in Myopathies
Source: Adv Sci (Weinh). 2026 Oct 31:e78533. Online ahead of print. doi: 10.1002/advs.78533 (PMC13723716; doi:10.1002/advs.78533)
Supplement: Supplementary file 3 [file ADVS-9999-e78533-s003.docx]

**Table S1.** Detailed information of the RUNX1 coding sequence (5’-3’) cloned into the overexpression plasmid pcDNA3.1(+).

| **Gene** | **Coding sequence (5’-3’)** |
| --- | --- |
| RUNX1 | ATGGCTTCAAACAGCGTATTTGATTCACTTCCTTCTTACCAGCACTTCTTGAGAGATACCAGCACCAGCCGCCGCTTCACGCCGCCCTCCACCGCGCTGAGCCCGGGGAAGATGAGCGAGCCGCTGCCGCTGCCCGGCGCCGAGCACAGCGGAGCGCTGGCGGGGAAGCTGCGCGCCGCCGACCGCAGCATGGTGGAGGTCCTGGCGGACCACCCCGGGGAGCTGGTGCGCACCGACAGCCCCAACTTCCTCTGCTCCGTGCTGCCCACCCACTGGCGCTGCAACAAGACGCTGCCCATCGCCTTCAAGGTGGTGGCCTTGGGAGACGTACCCGATGGGACGCTGGTCACGGTGATGGCTGGAAACGATGAGAACTACTCTGCAGAGCTCAGAAATGCGACGGCTGCCATGAAAAACCAAGTGGCGAGGTTCAACGACCTCAGATTTGTGGGTAGAAGTGGAAGAGGGAAAAGCTTCACACTGACTATCACAGTTTTCACAAACCCGCCACAAGTAGCCACGTACCACAGAGCCATCAAGATAACGGTGGATGGACCACGCGAACCCAGAAGACATCGTCAGAAACTAGATGAGCAGACAAAGCCCGGGAGCTTGTCCTTTTCAGAGCGCCTGAGTGAACTGGAGCAGTTGCGTCGTACGGCCATGAGGGTCAGCCCACACCACCCAGCCCCCACACCCAACCCACGAGCCTCCTTGAACCACACCACTGCTTTTAACCCTCAGCCTCAGAGTCAGATTCAGGACACGAGGCAAGTGCAGCCGTCCCCGCCATGGTCCTACGACCAGTCCTACCAGTACCTGGGCTCCATCGCGACTCCCTCGGTGCACCCTGCCACGCCGATATCGCCTGGCAGGGCCAGCGGCATGACGTCCCTCACCGCAGAGCTTTCCAGCCGCCTCTCAGGTGCTTCTGACTTGACGGCATTCAGTGACCCCAGAGTGGGGATCGACCGCTCCTTCTCCGCGCTC |

|  | CCTTCCATTTCTGATCCTCGGATGCACTACCCGGGAGCGTTCACCTACACGCCAACACCCGTCAGTTCGGGGATAGGGATCGGTATGTCTGCTATGTCCACGGCCACCAGATACCACACTTACCTCCCACCTCCATATCCCGGTTCCTCTCAGGCCCAAGGCAGCCCATTCCAGACCAGCTCACCTTCGTACCACCTCTACTACGGAACCTCTGCCGGGTCCTATCAGTTCTCCATGATCTCAGGAGGAGACCGGTCCCCCCCGCGCATCCACCCCCCTTGCACCAATGCTTCCACAGGATCGACACTCCTCAACCCCAACCTTCCCAACCAAAGCGATGTGGTTGAGGCAGAAGGGAGCCACAGCAACTCCCCAACCAACATGGGTACCACGGCAAGGCTAGAGGAAGCGGTTTGGAGGCCATACTGA |
| --- | --- |

**Table S2.** Detailed information of siRNA sequences.

| **siRNA** | **siRNA direction** | **siRNA sequence (5’-3’)** |
| --- | --- | --- |
| si-566 | sense | CCAGAAGACAUCGUCAGAAACUAGAdTdT |
|  | antisense | UCUAGUUUCUGACGAUGUCUUCUGGdAdT |
| si-572 | sense | CGUCAGAAACUAGAUGAGCAGACAAdTdT |
|  | antisense | UUGUCUGCUCAUCUAGUUUCUGACGdAdT |
| si-577 | sense | GACAUCGUCAGAAACUAGAUGAGCAdTdT |
|  | antisense | UGCUCAUCUAGUUUCUGACGAUGUCdAdT |

**Table S3.** Primer sets used for quantitative RT-PCR analyses.

| **Gene name** | **Accession number** | **Product size** | **Direction** | **Sequence (5’-3’)** |
| --- | --- | --- | --- | --- |
| *COL1A1* | XM_025144131.1 | 167bp | Forward | GAACCCCAAGGAGAAGAAGC |
|  |  |  | Reverse | TCTTGCAGTGGTAGGTGACG |
| *FN1* | NM_001198712.1 | 98bp | Forward | ACCTGCCTGATACTGCTACCTCTG |
|  |  |  | Reverse | ACTCTCCTGATGTTCCTCCACTGC |
| *ACTA2* | NM_001031229.1 | 176bp | Forward | CACCCAACTCTGCTGACTGA |
|  |  |  | Reverse | ACACCATCCCCAGAGTCAAG |
| *GAPDH* | NM_204305.2 | 134bp | Forward | CAGAACATCATCCCAGCGTCCAC |
|  |  |  | Reverse | CGGCAGGTCAGGTCAACAACAG |
| *RUNX1* | NM_001396968.1 | 101bp | Forward | CCTCAGCCTCAGAGTCAGATTCAG |
|  |  |  | Reverse | AGGTACTGGTAGGACTGGTCGTAG |
| *PCNA* | NM_204170.3 | 217bp | Forward | GTGCTGGGACCTGGGTT |
|  |  |  | Reverse | CGTATCCGCATTGTCTTCT |
| *CDK2* | NM_001199857.2 | 171bp | Forward | CCAGAACCTCCTCATCAAC |
|  |  |  | Reverse | CAGATGTCCACAGCAGTC |
| *CyclinD1* | NM_001396513.1 | 188bp | Forward | CAGAAGTGCGAAGAGGAAGT |
|  |  |  | Reverse | CTGATGGAGTTGTCGGTGTA |
| *MyOG* | NM_204184.2 | 89bp | Forward | TCCCGGAGCAGAGGTTTTAC |
|  |  |  | Reverse | CTCGGGACGCTCAGGAAATG |
| *MyOD* | NM_204214.3 | 73bp | Forward | CCCATGGAAATGACGGAGGG |
|  |  |  | Reverse | TGAAGCACGGGTCGTCATAG |
| *MyHC* | NM_001001302.2 | 174bp | Forward | GCCCAGAACAAGCCTTTTGA |
|  |  |  | Reverse | CTTGGGAGGGTTCATGGAGT |
| *TGFβ1* | XM_046937641.1 | 109bp | Forward | ATGAGTATTGGGCCAAAG |
|  |  |  | Reverse | ACGTTGAACACGAAGAAG |
| *TGFβ3* | NM_205454.1 | 227bp | Forward | TCTTTACATTGACTTCCGAC |
|  |  |  | Reverse | TCCTCCCAACATAGTACAAG |
| *TGFBR1* | NM_204246.2 | 85bp | Forward | TCCCAAACAGATGGCAGAGC |
|  |  |  | Reverse | CTAGCAGCTCCGTTGGCATA |
| *TGFBR2* | NM_205428.2 | 88bp | Forward | TATGCCTCCTGGAAGACCGA |
|  |  |  | Reverse | GCTCCTCTGCGGTCAAGAAT |
